# Vibrational resonance, allostery, and activation in rhodopsin-like G protein-coupled receptors

**DOI:** 10.1038/srep37290

**Published:** 2016-11-16

**Authors:** Kristina N. Woods, Jürgen Pfeffer, Arpana Dutta, Judith Klein-Seetharaman

**Affiliations:** 1 Physics Department, Carnegie Mellon University, Pittsburgh, PA 15213, USA; 2 Institute for Software Research, Carnegie Mellon University, Pittsburgh, PA 15213, USA; 3 Department of Structural Biology, University of Pittsburgh School of Medicine, Pittsburgh, PA 15260, USA; 4 Warwick Medical School, University of Warwick, Coventry CV4 7AL, UK

## Abstract

G protein-coupled receptors are a large family of membrane proteins activated by a variety of structurally diverse ligands making them highly adaptable signaling molecules. Despite recent advances in the structural biology of this protein family, the mechanism by which ligands induce allosteric changes in protein structure and dynamics for its signaling function remains a mystery. Here, we propose the use of terahertz spectroscopy combined with molecular dynamics simulation and protein evolutionary network modeling to address the mechanism of activation by directly probing the concerted fluctuations of retinal ligand and transmembrane helices in rhodopsin. This approach allows us to examine the role of conformational heterogeneity in the selection and stabilization of specific signaling pathways in the photo-activation of the receptor. We demonstrate that ligand-induced shifts in the conformational equilibrium prompt vibrational resonances in the protein structure that link the dynamics of conserved interactions with fluctuations of the active-state ligand. The connection of vibrational modes creates an allosteric association of coupled fluctuations that forms a coherent signaling pathway from the receptor ligand-binding pocket to the G-protein activation region. Our evolutionary analysis of rhodopsin-like GPCRs suggest that specific allosteric sites play a pivotal role in activating structural fluctuations that allosterically modulate functional signals.

G protein coupled receptors (GPCRs) are seven transmembrane (TM) helical bundles comprising the main chemical sensors capable of responding to a wide range of signals including hormones, neurotransmitters, cytokines, smells and light^1^,^2^,^3^, making them a premier pharmacological target. Recently, the number of solved GPCR structures^3^,^4^,^5^,^6^,^7^,^8^,^9^ has sky-rocketed providing detailed insight into their conformational states. Nonetheless, the mechanisms of receptor activation by their ligands are still not fully understood as these are intimately tied with protein and ligand dynamics^10^,^11^,^12^. It is generally accepted that GPCRs utilize an allosteric signaling mechanism to move the ligand binding signal across the membrane^13^,^14^,^15^. Allostery in proteins enables the activity of one site in a protein to modulate function at another spatially distinct region. In GPCR’s ligands typically bind in the TM domain near the extracellular (EC) surface or even in the EC domain, a signal which is transmitted through conformational change in the TM domain to alter the structure in the distant cytoplasmic (CP) domain. The CP side is the location of interaction with the G protein, which transmits the activation signal to downstream targets. The conformational change in the CP domain also induces an opposing signaling cascade leading to de-activation, initiated by phosphorylation of the C-terminus by a kinase, and binding of arrestin to the phosphorylated receptor. The structure of rhodopsin in complex with peptides representing the G protein^16^,^17^,^18^, and with arrestin^19^ have been solved, providing insight into the details of the interfaces between these large signaling complexes.

How is allosteric signal transmission realized? It has been proposed that local structural fluctuations (LSFs)^20^,^21^,^22^ play a major role in allostery in proteins and recent computational investigations with rhodopsin^23^,^24^ have indeed supported the presence of a pre-organized network of connections that link allosteric sites via localized protein interactions to distant, functional sites. This pre-organized network of interactions is present in the *inactive state* of the receptor^25^ and would provide a mechanism for activation (formation of Metarhodopsin II (Meta II) in the case of rhodopsin) in which concerted structural changes can spread to distant sites in the network.

Pharmacology of GPCRs is complex involving ligands that can be full, partial or inverse agonists and antagonists, and they have evolved such that each receptor can potentially control multiple intracellular signaling pathways^26^,^27^,^28^. This variability is supported by an ensemble of receptor conformations^29^,^30^,^31^ that enables dynamic adaptation. A dominant pathway emerges from this dynamic conformational landscape only after a specific event such as ligand binding or changes in EC conditions shift the ensemble of the already existing pathways, which in turn can trigger a specific intracellular response or a set of responses.

In this work our interest is to identify the intermolecular interactions^32^ that form the basis for the allosteric signal propagation in GPCRs. Computational approaches alone^23^,^24^,^25^ cannot answer this question, and experimental approaches to study dynamics that are applicable to membrane proteins lack atomic detail^33^,^34^,^35^. Therefore, we have developed a combined computational-experimental approach for detecting, predicting, and elucidating the conformational diversity and molecular associations that lead to receptor activation.

The experimental part of our approach utilizes Terahertz (THz) spectroscopy, which (1) directly detects the internal fluctuations^36^,^37^ that define the intrinsic dynamics of proteins in the <100 cm^−1^ region of the spectrum and (2) is sensitive to local relaxations that reflect specific intramolecular and intermolecular thermally-induced fluctuations that are driven by external perturbations, such as ligand-binding, in the 100–200 cm^−1^ spectral region. The globally, correlated fluctuations (≤100 cm^−1^ spectral region) allow the protein to sample^38^,^39^ the ensemble of conformations that describe the free energy landscape of all possible protein conformations. Hence, their detection provides a means of determining how sampling of the available conformational substates shifts the distribution of populations. The 100–200 cm^−1^ region reports on more localized intermolecular associations that form the basis^40^ of allosteric signal propagation in proteins. These experimentally detected localized fluctuations are analogous to LSFs extracted from MD simulations. We therefore are able combine the THz measurements with MD simulations in order to connect the experimental data to specific residue and helical associations in the receptor structure.

We use the dim-light mammalian photoreceptor, rhodopsin, as our experimental model system for the GPCR family. While the vast majority of GPCRs are activated by small diffusible ligands, and covalent attachment of the ligand in the case of rhodopsin is unusual, overwhelming evidence from large numbers of prior studies demonstrate that the mechanism of activation is likely conserved^41^. Thus, while we cannot (yet) claim generality of the mechanism of activation proposed here for rhodopsin for other GPCRs, it provides a first glimpse into deep understanding of activation in the first GPCR structure investigated with this new approach. Furthermore, our coupling of the THz and MD results with evolutionary analysis of the rhodopsin-like GPCR family supports the view that the ligand-induced shifts in conformational ensembles and the consequent formation of pathway-selective receptor signaling are likely generic and will not rely on either ligand or G-protein specifics for formulation.

## Results and Discussion

### Combined experimental-computational approach to study allostery

**Figure 1:**
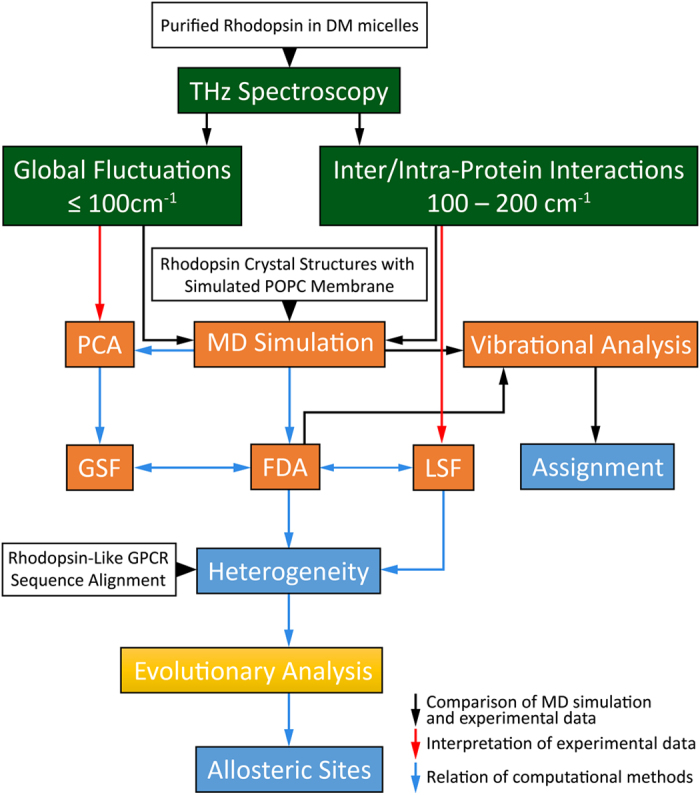
Overview of the methods used in this investigation. The black arrows indicate the MD simulation vibrational analyses methods that were used to assign the vibrational modes detected experimentally. The red arrows indicate the MD simulation methods that were directly used to interpret the experimental data. The blue arrows demonstrate how the computational methods relate to one another. In the chart PCA = principal component analysis, GSF = global structural fluctuations, FDA = force distribution analysis, and LSF = localized structural fluctuations.

Allosteric signal propagation takes place through complex dynamic fluctuations in protein intramolecular contacts and are thus difficult to predict by relying on sequence-based statistical methods even when they are merged with computational (MD) simulations^42^,^43^. We set out to fill this gap by developing a combined mathematical model-experimental approach, outlined in Fig. 1. Computed protein evolutionary networks are coupled with a molecular level view of allosteric effect and communication guided by experimentally detected protein associations. Explicitly, we used THz spectroscopy to directly probe the conformational fluctuations associated with the ensemble dynamics in rhodopsin in addition to the receptor interaction networks that promote allosteric interactions in the receptor 3-D structure. We complement our experimental results with MD simulation as a means of calculating and assigning vibrational modes associated with specific residue interactions and helical associations. We also use principal component analyses (PCA) from the MD simulations as a way of comprehending the nature and role of global dynamics in stabilizing specific signaling pathways in the photo-activated receptor, Meta II. We extract local (LSFs) and global structural fluctuations (GSFs) from the MD trajectories to gain a better understanding of the mechanism in which specific protein interactions form allosteric signaling pathways in rhodopsin and this is weighed by computing evolutionary conserved interactions from a sequence alignment of the rhodopsin-like family to determine distinctive allosteric sites that may contribute to the common functionality of the receptor family. Lastly, we use force-distribution analyses (FDA) from the MD trajectories to consider the role of the retinal in both distributing and propagating stress within the interior of the receptor due to the retinal interactions that take place within the ligand-binding pocket and to further comprehend the influence of the retinal dynamics on the global modes of the receptor.

### Conformational heterogeneity in dark-state rhodopsin

**Figure 2:**
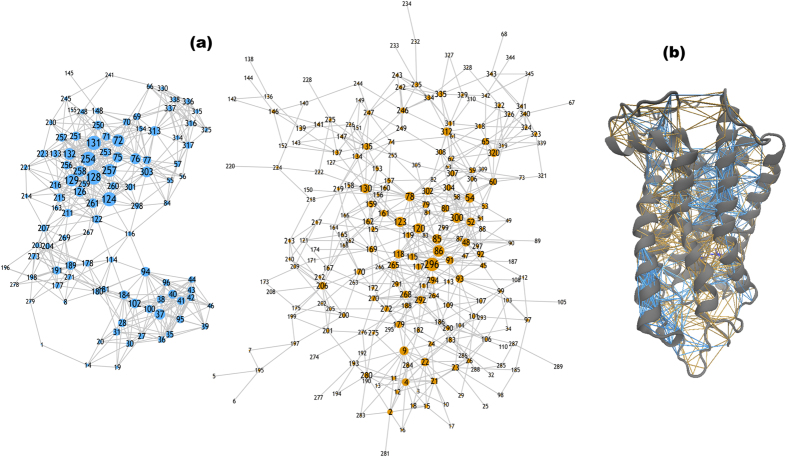
(**a**) Localized structural fluctuation (LSF) network of interactions from a MD simulation of rhodopsin in the inactive-state. In the network representation of the LSFs the nodes represent amino acid residues and the links between the nodes signify a localized interaction. The size of the nodes denote the number of interactions (connections) that a given node possesses. In the network representation of the LSFs we find that the connections of the inactive receptor are grouped into two separate communities that represent both inactive (blue) and active-like (orange) protein conformational fluctuations. (**b**) The corresponding fluctuations are mapped onto a 3-D cartoon representation of rhodopsin.

In rhodopsin, hydrogen-bonding (H-bonding) interactions between TM helices stabilize the visual pigment in an inactive conformation. However, an analysis of the local structural fluctuations (LSFs) from our MD simulation in Fig. 2 indicates that there is an equilibrium of both inactive and active-state protein conformational fluctuations (Figure S1) in the dark-state protein. This conformational heterogeneity suggests that rhodopsin samples a diverse set of functional structures in its inactive state. Furthermore, the heterogeneity of structural fluctuations detected also implies that there are potentially multiple allosteric pathways (Fig. 2b) that pre-exist in the dark-state receptor even before any activation event has taken place.

### Retinal torsional dynamics and the rhodopsin conformational ensemble

**Figure 3:**
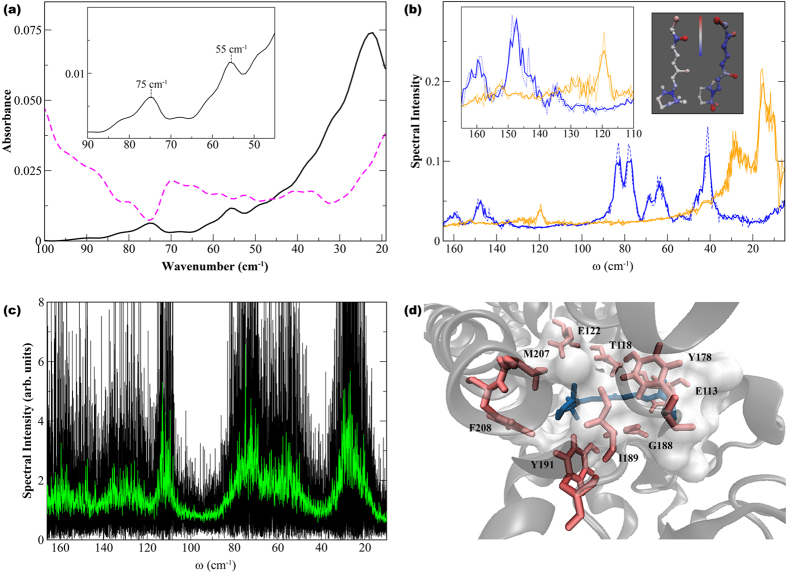
(**a**) The experimental THz spectrum of rhodopsin in the dark-state (black line) and in the light-activated state (magenta, dashed line) in the 20–100 cm^−1^ spectral region. The inset shows the 45–90 cm^−1^ spectral region of the dark-state protein. (**b**) The torsional spectrum of the retinal from rhodopsin MD simulations in the dark-state (blue line) and in the Meta II activated-state (orange line). The torsion of the retinal is defined by the angle created by the C5-, C9-, and C13- methyl groups. The inset on the right shows the mobility of the retinal motion from the (R) dark-state MD simulation and (L) Meta II simulation where blue represents less mobility and red more mobility. The inset on the left side shows the 110–165 cm^−1^ region of the calculated retinal torsional spectrum. (**c**) The Fourier Transform of the velocity autocorrelation function of protein residues surrounding the ligand-binding pocket in rhodopsin in the dark-state MD simulation. (**d**) Shows the residues used for the calculation of the velocity autocorrelation function spectrum in (**c**).

An analysis of the retinal torsional dynamics from the MD simulation of the inactive state receptor also points to a ligand that possesses conformational heterogeneity. Vibrational analyses of the retinal torsional fluctuations from the MD simulation of the inactive state reveal vibrational frequencies at approximately 85 cm^−1^, 65 cm^−1^, and 40 cm^−1^ in the ≤100 cm^−1^ region of the spectrum in Fig. 3b. A subsequent mapping of the interactions of the retinal with the receptor from a force-distribution analysis (FDA) reveals structural conformations of rhodopsin in both inactive and active-like conformations (Supplementary FDA and Figure S4). This supports the notion that the retinal ligand also possesses conformational heterogeneity.

In the dark-state, the retinal ring is highly distorted in its hydrophobic pocket and the retinal overall is constrained by the covalent linkage to Lys296 on helix 7, the Schiff base network and the salt bridge with Glu113 on helix 3^44^. The FDA of the dark-state shows that steric contacts between the ligand and residues in the immediate vicinity of the ligand-binding pocket excite structural fluctuations in the retinal that are closely connected with the dynamics of the entire protein (Figure S4 and Table 1). Analyses of the nature of the ligand-induced receptor fluctuations uncover two main conformations in the dark-state, (1) a dominant conformation (Figures S4a and S4c) where ligand-induced interactions promote a hinge-torsion in the global dynamics of the receptor that parallels the leading component from principal component analysis (PCA) of the dark-state from the MD simulation (Figure S4d), and (2) a minor conformation (Figures S4b,c) that arises from prominent, transient interactions of the receptor with the polyene chain methyl groups that resembles the ligand-induced interactions found in the crystal structure of Meta II^45^. In this case, the retinal-induced interactions induce an elongation torsion that moderately destabilizes contacts within the receptor core.

The dominant retinal ring conformation involves interactions with residues associated with both the oscillation of the Schiff base linkage in helix 3 and with weak ligand-induced forces with residues in the extracellular domain the promote stability in the retinal ligand-binding pocket in the dark-state of the receptor (Figure S4a). The minor retinal β-ionone ring conformation is associated with transient fluctuations that modify the ligand-receptor interactions such that the interaction with the C9- methyl group of the retinal has a much stronger interaction with hydrophobic core residues Gly120 and Gly121 on helix 3 (Figure S4b). Hence, the minor retinal-induced conformational orientation alters the packing interactions within the receptor hydrophobic core. Both orientations uncovered in this investigation are in line with previous MD simulations carried out by Lau *et al*. on dark-state rhodopsin^24^. In the previous study the torsional state of the retinal ring was found to occupy two distinct conformations: (1) a conformation in which the retinal C5-methyl group had strong contact with residues Thr118, Gly121, and Glu122 in the receptor core and (2) an orientation in which the β-ionone ring had stronger contact with residues Phe208, Tyr268, and Ala269 in the extracellular region of the receptor.

The FDA allows us to assign the retinal mode at 85 cm^−1^ in Fig. 3b as a torsional fluctuation while the peak at 65 cm^−1^ is associated with a chain torsion coupled with a retinal ring bending motion (Supplementary Figure S6 and Table 1). Both modes (at 65 and 85 cm^−1^) are related to rhodopsin in the dominant (inactive-type) conformation where both the polyene chain and the β-ionone ring are known to undergo constrained torsional twisting^46^. Likewise, the peak close to 40 cm^−1^ in the simulation spectrum of the 11-*cis* retinal in Fig. 3b involves a (polyene) chain torsional fluctuation in which the β-ionone ring movement is restricted by interactions with residues in helices 3 and 5. The 40 cm^−1^ retinal mode is a reflection of the ligand-induced interactions in the minor dark-state receptor conformation. The retinal peak at 65 cm^−1^ is coupled with a protein, global backbone torsion whereas the peaks at 85 cm^−1^ and 40 cm^−1^ are primarily linked with collective out-of-plane and in-plane protein side-chain fluctuations, respectively (Supplementary Figure S6).

### Experimental detection of the global modes of dark-state rhodopsin

In the <100 cm^−1^ region of the experimental THz spectrum of dark-state rhodopsin there is a prominent peak at approximately 25 cm^−1^ in the spectrum and additional, smaller peaks at 55 cm^−1^ and 75 cm^−1^ in Fig. 3a. There is also a shoulder in the spectrum at approximately 85 cm^−1^. The peak at 25 cm^−1^ is attributed to the global oscillation of polar side chains that are tied with the dynamics of the entire protein^47^, while the additional peaks are likely associated with collective helical oscillations^48^ that are at a similar frequency to the low-frequency peaks identified in the retinal torsional dynamics from the MD simulation in Fig. 3b.

### Vibrational analysis of receptor dynamics from MD simulation

Using vibrational analyses from the MD trajectories as a guide in interpreting the experimental data (Table 1), we find that the ~55 cm^−1^ mode in Fig. 3a is likely ascribed to a collective, torsional oscillation that involves backbone motion of all the TM helices and comprises the entire protein. The collective backbone motion connects the EC side of rhodopsin with the G-protein coupled region (Supplementary Figures S4d and S6). A comparison of the general vibrational analysis of the backbone motion to the largest PCA mode in the MD simulation supports the conclusion that the largest contribution to the backbone mode comes from residues surrounding the retinal-binding pocket but also includes smaller-amplitude oscillations from CP loop residues as well as EC loop residues that together, modulate the H-bonding network of interactions adjacent to the retinal (Supplementary Figure S4). These smaller-amplitude fluctuations taking place in the G-protein region extend the collective backbone fluctuation from the ligand-binding pocket out to the protein CP region (Supplementary FDA). The 55 cm^−1^ mode is closely coupled with the out-of-plane torsional oscillations of the β-ionone retinal ring as indicated by the PCA of the FDA. Interestingly, the polyene chain makes very little contribution to this low-frequency fluctuation (at 55 cm^−1^). Similarly, using the same vibrational analysis method we are able to tentatively assign the peak at 75 cm^−1^ in the experimental spectrum with collective oscillations of amino acid side-chains that are predominately clustered around the terminal portion of the retinal chain (C-13).

It is interesting to note that there is a 40 cm^−1^ retinal-receptor mode that is not prominent in the experimental room-temperature spectrum of inactive rhodopsin in Fig. 3a. In the previous section we identified a mode at this frequency that described retinal-rhodopsin interactions associated with a minor conformation in the receptor ensemble dynamics. Our calculation of the prominent dynamical fluctuations of specific residues residing in the retinal-binding pocket (Fig. 3c,d) also seems to support this supposition. The low-frequency spectrum (≤100 cm^−1^) of the distinct residues lining the ligand binding region contain peaks at approximately 25 cm^−1^, 55 cm^−1^, and 75 cm^−1^ but the 40 cm^−1^ mode is missing in Fig. 3c. The absence of the 40 cm^−1^ oscillation indicates that the fluctuation may be associated with a minor conformation that is not central in the equilibrium dynamics of the dark-state receptor.

### Retinal isomerization and shift in the conformational ensemble in Meta II

In Fig. 3b a plot of torsional oscillations of the retinal dynamics from the MD simulation in dark-state and Meta II demonstrates that relaxation of the retinal after isomerization shifts the peaks of torsion to lower frequency. Specifically, there are peaks at approximately 20 cm^−1^ and 10 cm^−1^ in the low-frequency vibrational spectrum that are both related to collective, polyene chain torsional fluctuations. From analyzing the retinal vibrational modes alone and then how they were coupled to the receptor dynamics, we are able to conclude that the peak at 10 cm^−1^ is related to a torsional fluctuation of the C-20 methyl group near the terminus of the polyene chain and the 20 cm^−1^ mode is associated with a collective chain-twisting oscillation.

The low frequency vibrational modes (≤100 cm^−1^) from the *all-trans* retinal from the Meta II MD simulation shown in Fig. 3b detail peaks that are visibly red-shifted when compared with the 11-*cis* retinal in the equivalent spectral region. Further examination from the FDA suggests that the shift in peak frequency reflects an increase in the dominance of methyl group fluctuations in the Meta II retinal dynamics that principally involve fluctuations of methyl groups (Supplementary Figure S5) of both the C-9 and C-13 atoms on the polyene chain^49^. An analysis of the origin of the retinal modes indicates that the mode at 20 cm^−1^ is coupled to the global dynamics of the more flexible active-state protein (Supplementary FDA). After photo-isomerization, the relaxation of torsional strain triggers an overall change in the dynamics of the retinal (Fig. 3b). Our FDA analysis of Meta II indicates that it is the conformational rearrangements in the receptor in response to the altered ligand dynamics that cause intermolecular contact variations in the vicinity of the retinal-binding pocket. These changes result in increased flexibility (Fig. 3) in the ligand chain^50^,^51^ while at the same time constraining the movement of the retinal β-ionone ring (Supplementary Figures S4–S6). Moreover, the increased flexibility of the retinal chain in Meta II is directly tied with the release of interhelical constraints (that are present in its inactive-state). These constraints underlie adjustments within the extended H-bonding network of residues surrounding the retinal-binding pocket. The changes in the ligand-induced protein interactions that take place after isomerization gives rise to agonist (*all-trans* retinal) binding that is more flexible than inverse agonist (*11-cis* retinal) binding in the retinal binding pocket. This has important ramifications on the flexibility of the entire protein. For instance, as a whole, we find that Meta II is far more flexible than the inactive protein (Fig. 3b and Supplementary Figure S6, and see discussion on the 110 cm^−1^ peak below).

Above 100 cm^−1^ the motions of the retinal from the MD simulation in Fig. 3b illustrates the effect of the more localized ring and chain torsions in the calculated vibrational spectrum. Assignments from the vibrational analysis (Table 2) determine that the peaks at approximately 160 cm^−1^ and 150 cm^−1^ are associated with the wringing, torsional oscillation of the β-ionone ring. From the FDA on both receptor states, we find that the retinal ring oscillations are interconnected with localized fluctuations of the retinal chain and the twisting motion of select, mobile regions of the polyene chain, respectively. Using FDA to analyze the coupling between retinal and receptor fluctuations in both the inactive and Meta II states of the receptor indicates that these more localized retinal motions are coupled with somewhat rigid fluctuations taking place in the protein helices and loops that serve as structural constraints in the receptor. The retinal torsional oscillations in Meta II reveal peaks at 120 cm^−1^ and 130 cm^−1^. The red-shift of the torsional peaks when contrasted with the dark-state receptor highlight an increased coupling of the C-9 and C-13 retinal methyl group dynamics with the helical fluctuations of Meta II.

### Experimental detection of global modes of Meta II

The low-frequency (<100 cm^−1^) experimental spectrum of Meta II in Fig. 3a differs significantly from the dark-state. In fact, there are no clearly resolved peaks in the equivalent region suggesting that the protein modes excited after isomerization are (1) not strongly-infrared active and/or (2) red-shifted to very low frequencies.

### Experimentally detected interaction networks in Meta II and dark-state rhodopsin

**Figure 4:**
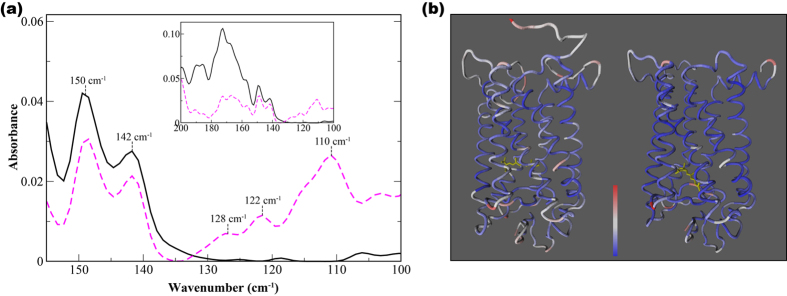
(**a**) The experimental THz spectrum of rhodopsin in the dark-state (black line) and light-activated state (magenta, dashed line) in the 100–160 cm^−1^ spectral region. The inset shows the entire 100–200 cm^−1^ spectral region of the experimental spectrum. (**b**) Cα− representation of rhodopsin showing the conformational mobility from the MD simulation of (R) dark-state and (L) Meta II activated-state of rhodopsin. Regions in blue represent areas of less mobility and regions in red more mobility.

In the >100 cm^−1^ region of the experimental spectrum we detect motions in rhodopsin that reveal more localized intermolecular interactions such as interhelical contacts as well as helical interactions with the solvent. For example in Fig. 4a, the peaks at approximately 150 cm^−1^ and 140 cm^−1^ in the dark-state spectrum are likely associated with interhelical and solvent-induced H-bonding interactions^52^. An analysis of the H-bonding dynamics of the intra- and interhelical fluctuations in both states of the receptor from the MD simulations reveal peaks at a similar frequency, further confirming our assignments (Table 1). From the analyses of the H-bonding dynamics from the MD simulation we determine that the receptor modes (at 150 cm^−1^ and 140 cm^−1^) are highly influenced by the retinal dynamics involving out-of-plane distortions that incorporate both the β-ionone ring and the polyene chain. Further, the helical fluctuations identified appear to be analogous to those detected in the MD simulation of the retinal at a slightly higher frequency in Fig. 3b. Above 160 cm^−1^ the experimental spectrum is dominated by modes that describe hydration and bulk water intermolecular interactions^53^,^54^.

In addition to the ~150 cm^−1^ and 140 cm^−1^ modes observed in the dark-state there are also additional peaks in the experimental spectrum at approximately 130 cm^−1^, 120 cm^−1^, and 110 cm^−1^ in the >100 cm^−1^ spectral region in the light-state receptor in Fig. 4a. The peaks at 130 cm^−1^ and 120 cm^−1^ are close in frequency to those uncovered in the retinal torsional spectrum of the MD simulation of Meta II in Fig. 3b. Additionally, we have identified peaks at similar frequencies in previous investigations on other proteins^48^,^55^. Hence, the vibrational analyses from the MD simulations carried out in this investigation combined with our previous studies on other proteins allow us to associate the experimentally observed peaks at about 120 cm^−1^ and 130 cm^−1^ in Fig. 4a with anharmonic, solvent-mediated fluctuations that couple to protein main-chain and backbone atoms, respectively^48^,^52^,^56^. It is interesting to note that both solvent-mediated modes oscillate distinctly from the solvent-induced modes observed in the experimental dark-state spectrum. This suggests that there is an additional, distinct class of water molecules interacting with Meta II that is not present and/or prominent in the dark-state protein. Further we determine that the localized fluctuations detected in Meta II are strongly coupled with torsional oscillations of methyl groups in the retinal polyene chain. Therefore, we assign the peak at 110 cm^−1^ in Fig. 4a with the torsional oscillation of methyl groups of solvent-exposed helices in the receptor. From earlier experiments on other proteins^48^,^52^ we have found that the experimentally determined 110 cm^−1^ peak is a general indicator of a flexible protein environment^48^. This supports our earlier notion that Meta II is more flexible than the dark-state receptor.

### Localized structural fluctuations (LSFs) and the formation of a dominant allosteric pathway in Meta II

**Figure 5:**
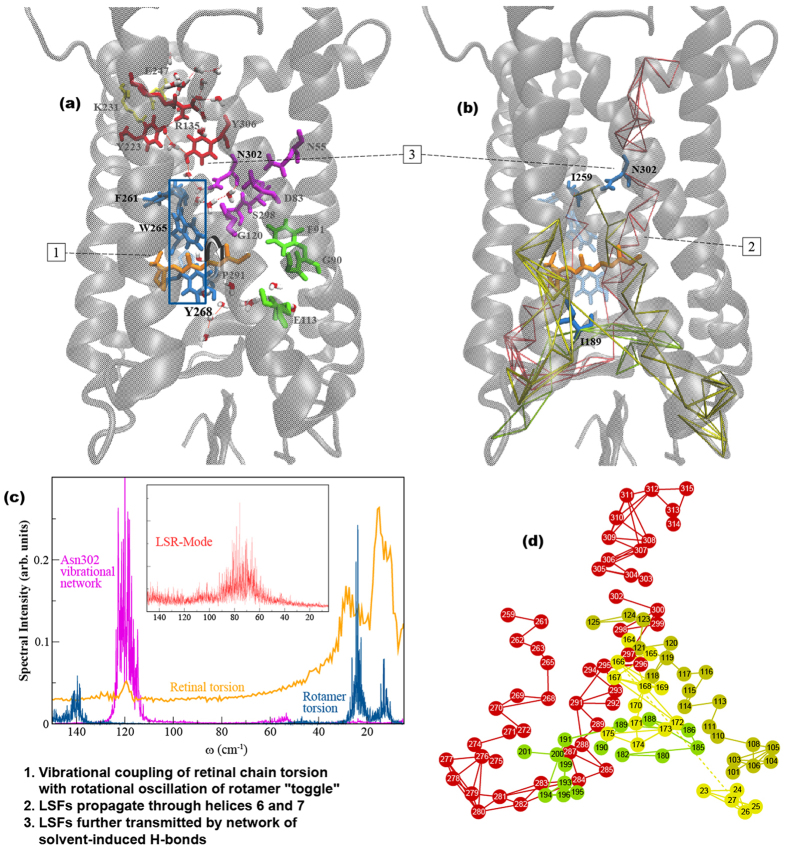
Analysis of the MD simulation of Meta II. (**a**) Conserved networks of H-bonds involving water molecules in rhodopsin taken from the literature^57^, highlighting the D(E)RY, CWxP, Schiff base-counter ion and NPxxY network motifs are shown in red, blue, green, and magenta licorice representation, respectively. The licorice representation of residues in the blue, rectangular box show the amino acids comprising the CWxP motif that is believed to convert between two different rotamer conformations in the inactive and active states. (**b**) Cartoon representation of Meta II showing the mapping of the LSFs from the MD simulation onto the protein 3-D structure. The different colors in the network mapping of the LSFs represent regions of correlated fluctuations. (**c**) The spectrum of the fluctuations from the MD simulation of Meta II showing the rotamer torsion (blue), torsional oscillation of the retinal (orange), vibrational fluctuation of the Asn302 network comprising the NPxxY motif (magenta), and the spectral signature of the localized solvent relaxation (LSR) mode (red) that couples with the conserved network of H-bonds. (**d**) A 2-D mapping of the LSFs from (**b**) where solid links correspond to strong interactions between residues and dotted links are associated with weaker connections. The numbers correspond to the stages of activation outlined at the bottom of the figure.

As described above, solvent-mediated H-bonding interactions appear to be an important constituent of the localized structural fluctuations that we detect experimentally. We therefore investigated the water-mediated H-bonding networks associated with highly conserved residues^57^ in rhodopsin and other rhodopsin-like GPCRs that are known to be involved with the functional mechanism of activation. Figure 5a displays all of the conserved networks of H-bonds involving water molecules in rhodopsin taken from the literature, highlighting the D(E)RY, CWxP, Schiff base-counter ion and NPxxY network. The localized structural fluctuations of water contacts in the conserved water motifs have been conjectured^58^ to play a prominent role in the transmission of activation signals in the family of receptors by mediating communication from the ligand binding pocket to the CP side where G protein binding and activation occur. We therefore analyze the structural fluctuations in these conserved water-mediated H-bond networks in more detail and consider them in relationship to the LSFs calculated in this investigation on rhodopsin.

In Fig. 5c,d, a graphical representation of the Meta II LSFs (from the MD simulation) clearly demonstrates that the magnitude of LSFs in the protein structure has been dramatically reduced in Meta II when contrasted with the inactive state protein in Fig. 2b. The LSFs in Meta II form a correlated set of (localized) fluctuations that connect helices 3, 4, and regions of the N-terminus as well correlated fluctuations of the β4 strand of EL2 that together function to stabilize the retinal in the *all-trans* configuration. Furthermore, the helical dynamics of the active-state induce LSFs in helices 6 and 7 that form a direct (allosteric) pathway from the EC side of rhodopsin to the CP region.

Local conformational fluctuations also modulate the coupling between the retinal and global structural fluctuations in Meta II. This is visible from the FDA of Meta II (Supplementary FDA) that shows that the flexibility of the ligand in the active-state is crucial for the development of localized, anharmonic fluctuations that form the basis for allosteric signal propagation. An analysis of the LSFs from the Meta II MD simulation in Fig. 5c show that changes initiated in the H-bonding network of residues directly adjacent to the photo-excited ligand mediate localized, collective fluctuations in areas incorporating the EC loop 2 (EL2) along with the EC ends of helices 4–6. The modification of helical contacts leads to steric clashes between the *all-trans* retinal and residues in helix 5 that result in the subsequent displacement of the retinal β-ionone ring away from Trp265 (Trp6.48 in the Ballesteros-Weinstein notation^59^) in helix 6. The shift of the retinal ring away from Trp265 initiates an outward rotation of helix 6 on the CP side. The outward rotation of Trp265 has been identified as the key to receptor activation^60^. From our analysis we find that coupling of the flexible, retinal chain torsional oscillations with the localized rotational oscillation (rotamer “toggle”)^61^ consisting of Phe261, Trp265, and Tyr268 that overlap with the CWxP motif on helix 6 (Fig. 5a) forms the underlying mechanism for allosteric pathway formation in Meta II. The development of the thermally activated process consisting of the ligand and the localized rotational mode is facilitated by the disruption of the dark-state extended H-bond network consisting of conserved interhelical constraints. We determine that through the disruption of these constraints, the localized rotational fluctuation is propagated through helices 6 and 7 via LSFs and the signal is further transmitted to the CP side of the protein by means of a conserved network of solvent-induced H-bonding interactions. This creates a defined route comprised of correlated, anharmonic fluctuations connecting the retinal binding-pocket dynamics with oscillations taking place in the G-protein binding region of rhodopsin as shown in Fig. 5c.

The FDA of Meta II also confirms that the new contacts established after isomerization stabilize the active-state receptor (Supplementary FDA). These newly formed interactions also make other conformations within the ensemble less accessible. In effect, light activation has shifted the equilibrium of the conformational ensemble toward a specific active conformation and changes in specific intermolecular interactions have been adjusted to stabilize it^62^. As a result, Meta II is comprised of a more homogenous ensemble of structures and consequently a single, dominant allosteric pathway linking the retinal-binding pocket to the protein CP domain^23^.

From the examination of the overlap of the Meta II LSFs with the GSF (Supplementary Signal propagation and GSFs) we find that the relaxation of the retinal after isomerization not only modifies its dynamical motion but the observed changes underlie alterations in the protein intrinsic fluctuations. It is likely that we observe these changes in the receptor global fluctuations experimentally when contrasting the dark and light-state of rhodopsin in Fig. 3a. In this narrative, the retinal has a decisive role in the conformational selection mechanism. The ligand induced-protein fluctuations induce allosteric changes in the receptor global modes and the change in receptor dynamics reflects the shift of the relative populations of conformational states sampled within the ensemble.

### The role of coupling of localized vibrational modes in the dynamic allostery of Meta II

The dynamical analysis of the Meta II LSFs are shown in Fig. 5b and a mapping of the LSF components onto the Meta II structure is shown in Fig. 5c. In Fig. 5b we can detect a *vibrational* resonance between the retinal methyl fluctuations, the localized rotational oscillations of the rotamer, and the conserved, solvent-mediated H-bond networks that are activated with light isomerization. One network in particular stands out, namely that associated with Asn302. It serves as an intermediary between the coupled networks as can be seen in the mapping of the LSF onto the structure in Fig. 5c. Structurally, it stabilizes the infrastructure for a collective, fluctuational focal point that oscillates distinctly from the rest of the receptor associations. This distinctive resonance of correlated fluctuations forms a coherent pathway of allosteric communication in the receptor 3-D structure (steps 1–3, Fig. 5). This analysis further supports our above conclusion that the peaks detected in the experimental THz spectrum in Fig. 4a at 120 cm^−1^ and 130 cm^−1^ reflect the anharmonic fluctuations associated with the allosteric interactions in Meta II. These anharmonic fluctuations do not appear to be present in the dark state receptor.

It is also worth mentioning that a vibrational analysis of the significant interactions forming the LSFs in Meta II from the MD simulation has uncovered a distinct class of water molecules associated with the conserved H-bonding network that forms part of the allosteric interface. The water molecules forming the network oscillate at 80 cm^−1^ (Fig. 5b) and are located close to the surface of residues identified as conserved micro-switches in rhodopsin. Previous experimental^63^ and computational^53^,^64^ investigations on protein hydration water have identified a peak at a similar frequency and in these instances it was described as a localized, water relaxation mode that fosters the propagation of collective, vibrational oscillations in hydrated proteins. The localized solvent-relaxation (LSR) mode would be expected to be a negligible component of the hydration water in rhodopsin and it is unlikely that such a mode would be detected experimentally. Nonetheless, we propose that these water molecules form an integral part of the allosteric network.

### The possible origin of long-range allostery in rhodopsin

Asn302 (Asn7.49) of the conserved NPxxY motif is located at the intracellular end of TM VII. As described above, it has been identified as a constituent of the conserved network of H-bonds in rhodopsin^65^ and other rhodopsin-like proteins^26^,^66^ that provide structural constraints in the dark-state and dynamically rearrange after photo-isomerization in the transition to the active Meta II state (Fig. 5c).

The results from this investigation suggest that the residue may also have a more integral role in connecting the ligand dynamics with the protein collective fluctuations that are involved with intracellular signaling. We find that Asn302 in rhodopsin functions as important mediator in the formation of the extended allosteric network (Fig. 5c,d). It links the collective helical oscillations that trigger activation with the conserved H-bonding network of fluctuations that extend the signal to the CP interface.

Asn302 also has a central role in the ligand-induced shift in the conformational ensemble in rhodopsin that takes place after isomerization. In previous sections we have demonstrated that the ligand motion is coupled with the conserved network of H-bonding interactions and conversely, the protein motion with the retinal (Fig. 5). Therefore changes in intermolecular interactions in the extended allosteric network would result in a shift in the equilibrium of the population of accessible conformers. Hence, Asn302 represents an important component in the allosteric network in that it is has the capacity to modulate the extended allosteric environment.

**Figure 6:**
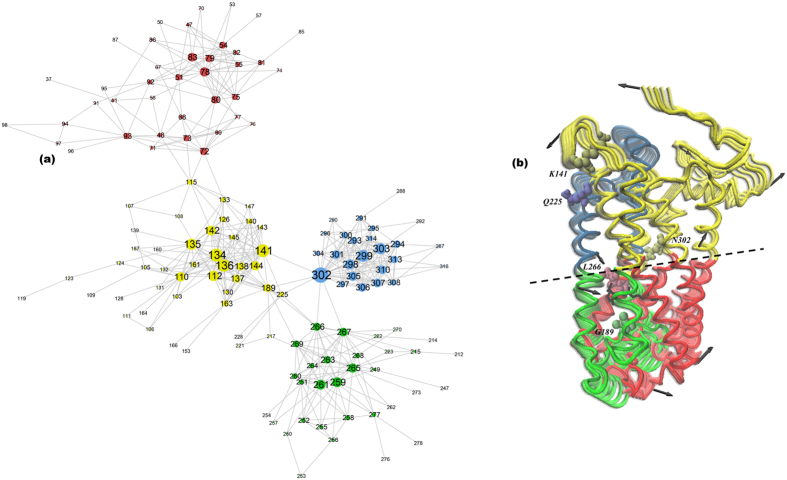
(**a**) MSA of the rhodopsin-like family of proteins broken into communities with each community containing different colors. In communities, nodes within the same community are highly connected and communicate with one another efficiently in a localized manner, whereas nodes in different communities have fewer connections and require intermodular edges (or links) for forming long-distance communication. The nodes represent amino acid residues, the links are the connections between the residues, and the size of the nodes denotes the number of connections (links) to other nodes. The reference structure for the MSA is bovine rhodopsin (opsd_bovin) with pdb ID 1u19. (**b**) Cα− atom representation of rhodopsin showing the global hinge-like elongation torsion of Meta II from the dominant PCA mode (PCA1) from the MD simulation. The global torsion has been divided into structural domains where each domain is colored separately. The line shown depicts the bisector region of the separate domains.

Earlier studies on rhodopsin have indicated that there is an evolutionarily conserved network of residues that communicate the pathway of signal transmission in the general rhodopsin-like family^67^. From a multiple sequence alignment (MSA) analysis carried out on rhodopsin-like proteins in this work (Fig. 6a) we find that Asn302 has a central position in the network of densely connected residues. It is the most connected amongst residues, even higher than Glu134/R135 of the D(E)RY motif (Figure S9). This suggests that its role in the signal propagation mechanism in the protein family may be significant. It has links to a number of loops and functional residues that allude to its importance in coupling a structural arrangement of connected loops that correlate the movements of EL2 near the retinal-binding site with specific residues in helices 3–7 (Supplementary Signal propagation and GSFs). The correlated movements are associated with helical rearrangements and the subsequent disruption of H-bonding constraints that are required for receptor activation^68^ and have also been shown to contribute to the binding surfaces involving tertiary contacts^69^ of both rhodopsin and G_t_.

The MSA also reveals that Asn302 has high coevolution propensity (Figure S10) signifying that it may have a prominent role in substrate binding or recognition^70^. It forms links *between* a number of different network communities. In general, highly coevolved residues that serve as “linkers” between different network communities like Asn302 have been shown to facilitate enhanced long-range signaling in the network of connected residues^56^,^71^. It has been proposed that through these linker residues a signaling pathway is modified when a perturbation is introduced. In other words, high coevolution value linker residues are effective in altering signaling pathways by establishing (or eliminating) channels of long-range communication within the protein network. It provides key connections between the individual components of the allosteric signaling pathway. In this work we find that the position of Asn302 in the greater rhodopsin-like MSA implies that the residue is associated with a structural signature that may be linked with a general mechanism for activation in the rhodopsin-like family of receptors rather than attributed to a specific mechanism associated with G-protein recognition or binding.

### Signal propagation and globally correlated structural fluctuations in rhodopsin

Following the pathway of mechanical stress that is propagated within the interior of the receptor due to retinal isomerization with FDA, we find that activation leads to a global hinge-like, elongation torsion in rhodopsin (Supplementary FDA and Supplementary Signal propagation and GSFs). The torsion conjointly releases constraints associated with the inactive receptor and stimulates interactions that support the formation of a dominant allosteric signaling pathway in Meta II. The global torsion is communicated as large changes in main chain torsional angles involving a small number of residues in a localized region. For example, residues that form part of the CWxP rotamer, especially Pro267 (Pro6.50), located near the central hinge region in Fig. 6b promote movement in TM6 on the opposite side of the hinge. The localized rotational motion of the Trp265 (Trp6.48) rotamer is further correlated with fluctuations in both EC (EL2) and CP loops (CL2 and CL3) that foster oscillations in conserved helical regions^72^ (Supplementary Figure S10) in the rhodopsin-like family that are known to be involved with activation. Interestingly, the dynamics of Asn302, which sits at the midpoint region connecting the separate domains of the global hinge torsion, provides the coupling point of a conserved, extended H-bonding network of residues^58^,^73^ (comprised of residues within the same MSA network community as Asn302 as well as residues in different, connected communities) with the LSR mode. The extended dynamics of the hinge residue coupled with the LSR mode creates an amplified signal-propagating element in the protein 3-D structure. Together, the strongly correlated rotational motions involving the centrally located hinge point residue (Asn302 in rhodopsin), the LSR mode, and the conserved rotamer define a relaxation mechanism on a local level that ultimately unites regions associated with the long-range allosteric interactions that are triggered with activation.

### Rhodopsin bound to arrestin, heterogeneity of retinal conformations, and multiple signaling pathways

**Figure 7:**
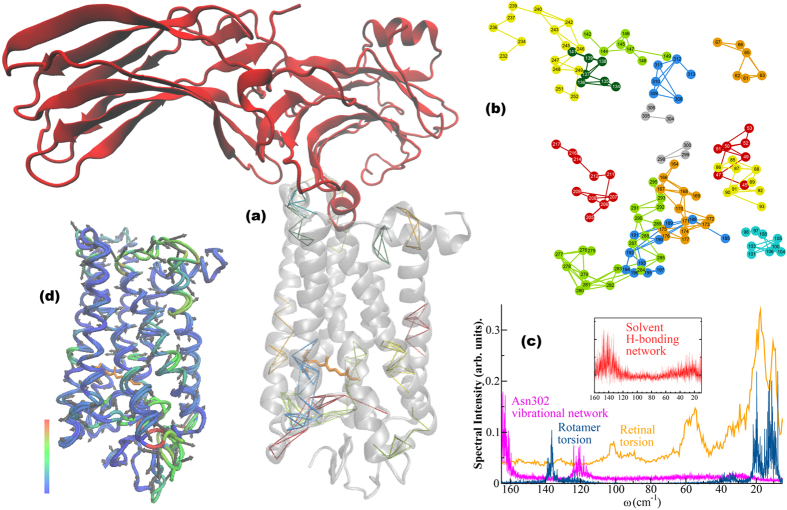
(**a**) Cartoon representation of active-state rhodopsin bound with arrestin showing the mapping of the LSFs from the MD simulation onto the rhodopsin 3-D structure. The different colors in the network mapping of the LSFs represent regions of correlated fluctuations. (**b**) A 2-D mapping of the LSFs from (**a**). (**c**) The spectrum of the fluctuations from the MD simulation of the arrestin-bound rhodopsin showing the rotamer torsion (blue), torsional oscillation of the retinal (orange), vibrational fluctuation of the Asn302 network comprising the NPxxY motif (magenta). The inset in (**c**) shows the spectral signature of the solvent H-bonding fluctuations (red) that couple with the conserved network of water-mediated H-bonds in rhodopsin. (**d**) Illustration of the dominant PCA mode (PCA1) of rhodopsin from the MD simulation of rhodopsin bound with arrestin where the areas in blue show regions with less mobility and the areas in red show regions with more mobility.

To test the significance of our findings for rhodopsin signal transduction, we extended our LSF analysis to the recently determined structure of the arrestin-rhodopsin complex^19^. The comparison with our Meta II analysis allows us to extract the localized fluctuations that are induced by arrestin binding (Fig. 7a,b). We find that the binding of arrestin disrupts the highly conserved functional interactions that form structural motifs that stabilize Meta II. These interactions include Arg135 – Tyr223 which stabilizes the TM3-TM5 interface and the Lys231 – Glu247 salt-bridge that forms the CP TM5-TM6 connection. The Asn302 network of interactions is also disrupted with arrestin binding leading to a sampling of two distinct H-bonding environments on the time scale of the simulation. The peak in the Asn302 network at approximately 165 cm^−1^ in Fig. 7c involves interactions of molecules involved with strong interhelical H-bonding and the smaller peak at 120 cm^−1^ represents a smaller component of molecules that are analogous to the H-bonding contacts found in Meta II not bound with arrestin (Fig. 5b). The heterogeneity within the Asn302 network is further confirmed from the analysis of the water molecules incorporated within the cluster of interactions (Fig. 7c inset) that form the extended network of H-bonds of the molecule. The spectrum of water molecules reveals two peaks. The peak at about 145 cm^−1^ describes water molecules integrated within the rigid interhelical H-bond fluctuations of the receptor, while the smaller shoulder in the spectrum at approximately 20 cm^−1^ describes water molecules with a prominent rotational character. The latter group reflects those dynamic water molecules that are able to move more freely within the receptor solvation shell in the vicinity of conserved network of H-bonding interactions. The cluster of anharmonic solvent H-bonding molecules that form part of the allosteric interface in Meta II at 80 cm^−1^ (LSR mode) is also absent in the arrestin-bound rhodopsin complex further signifying the splintered long-range connections in the receptor.

It is interesting to point out that the weakening of interhelical interactions, particularly those involved with the Asn302 H-bonding cluster of interactions, directly results in changes in the network of interactions surrounding the ligand-binding pocket. The coupling between distinct components of the allosteric network (Fig. 5c,d) is disrupted with arrestin binding leading to a disconnect between the dynamical interactions taking place within the retinal ligand-binding pocket and the network of interactions that lead toward the G-protein binding region of the receptor (Fig. 7b). For example, the break-down of stabilizing interactions in the functional microdomain regions of Meta II is also the source of increased retinal mobility in the ligand-binding pocket (Fig. 7c,d). Residues such as Tyr178 and Ile189 that normally constrain the β-ionone ring in Meta II have increased mobility as a result of arrestin binding. Moreover, the modulation of the ligand-binding pocket residues is directly tied with the increased structural flexibility of the retinal (Fig. 7c). In this investigation we have conjectured that the ability of the retinal to assume multiple conformational states is intertwined with the generation of multiple, distinct signaling pathways in both the inactive and active receptor. From this point of view, it is interesting to point out that arrestin does promote a series of fragmented small-scale structural fluctuations in Meta II (Fig. 7a,b and d). None of these induced-fluctuations appear to form long-range correlated fluctuations that would create a distinct signaling pathway from the retinal ligand-binding pocket to the arrestin binding region. This suggests that in addition to its assumed role of providing steric hindrance to the binding of the G protein, arrestin binding fragments the activation signal in rhodopsin itself.

## Conclusions

In this investigation we use a combined experimental and computational approach to examine the conformational ensemble dynamics and the consequent formation of allosteric interactions in rhodopsin with the aim of gaining a molecular level understanding of activation. We find that the dynamics of the retinal are directly correlated with shifts in the equilibrium of the population of states of the protein conformational landscape. In particular, the detected coupling of the ligand dynamics to the protein low frequency modes^64^,^74^,^75^ indicates that the retinal motion is directly tied with the overall protein configuration during the photo-isomerization process. In the hydrophobic ligand-binding pocket the retinal makes contacts with various residues that subsequently alters the dynamical ensemble of the accessible conformations. Hence, the ligand-induced protein interactions have a strong influence on the potential stabilization/destabilization of the functioning protein conformations.

We also determine that the local changes to structural constraints and reorganization of an extended H-bonding network involving conserved residues and water molecules mediate allosteric communication in Meta II. The conserved protein-solvent H-bonding interactions construct a pathway of interconnected fluctuations that oscillate distinctly from the rest of the protein. This creates a coherent route from the ligand-binding pocket out to the CP region where G-protein activation occurs.

These networks of conserved interactions represent adaptable communication pathways that change with activation. In the dark-state the specific network of H-bonding interactions act to stabilize the protein. In the active-state they arbitrate the structural plasticity linked with ligand-induced shifts in the conformational ensemble. The localized conformational fluctuations that are triggered with isomerization couple with the retinal motion. Together the active-state protein-ligand dynamics, modulated by a co-evolutionary network that regulates long-range communication in the rhodopsin-like GPCR family, steer the sampling of conformational states within the pre-existing ensemble. The associated dynamics alter the equilibrium of the populated states, which consequently is central in the formation of a single, major allosteric pathway in the protein 3-D structure. This ultimately links the EC side of the protein with that of the CP region. The long-distance concerted structural changes that arise in response to activation in rhodopsin may also hint at a common network of interactions in the mechanism of activation of class A GPCRs.

## Materials and Methods

### Sample Preparation

Rhodopsin samples were purified in detergent micelles composed of dodecyl maltoside (DM). The choice of DM as a detergent is justified because the conformational changes in rhodopsin in DM are virtually identical to those seen in liposomes^76^. Rhodopsin samples were obtained through transient transfection or from stable cell lines. Transient transfection of COS-1 cells was carried out as described^77^, with the exception that the cells were harvested 72 h after transfection. Tetracycline inducible HEK293S stable cell lines were established as described previously^78^. Both types of cells were solubilized with 1% (w/v) DM for one hour and the proteins were purified by 1D4 immuno-affinity chromatography in 0.05% DM as described^79^. Briefly, after solubilization of the cells, the suspension was centrifuged for 30 min at 35,000 rpm and 4 °C. The supernatant was mixed with 1D4 Sepharose beads (approximate binding capacity of 1 μg rhodopsin/μl of resin) for at least 6 h at 4 °C. The resin was then washed with 50 bed volumes of 0.05% (w/v) DM in PBS followed by 10 bed volumes of 0.05% (w/v) DM in 2 mM Na_2_HPO_4_/NaH_2_PO_4_ (pH 6.0). WT and mutant proteins were eluted with 70 μM C-terminal nonapeptide (TETSQVAPA) in 0.05% (w/v) DM in 2 mM Na_2_HPO_4_/NaH_2_PO_4_ (pH 6.0). The initial concentration of the sample was determined by UV absorbance and subsequently diluted to a concentration of 200 μm in preparation for the THz spectroscopy experiments.

The rhodopsin samples used in the THz spectroscopy experiments were prepared by allotting 20 uL of the prepared sample onto a custom ordered diamond transmission window (Specac Co., United Kingdom). Excess water from the solution was initially removed by applying a low, steady flow of N_2_ gas over the sample droplet for approximately 3 minutes. The resulting sample was subsequently rehydrated by equilibrating the dried-off sample in a vacuum sealed container with the vapor pressure of a saturated salt solution at 20 °C for a minimum of 3 days. A relative humidity (RH) of 97% was obtained from the vapor pressure of a saturated K_2_SO_4_ solution^80^. The prepared rhodopsin sample was subsequently placed in a sealed transmission cell consisting of two diamond window substrates and a saturated salt solution was placed at the bottom of the cell to ensure that hydration was maintained throughout the experiment.

### THz Spectroscopy Experiments

The dark-state rhodopsin experiments were performed under dim-red light conditions and photo-isomerization was triggered with visual light excitation. The THz spectroscopy experiments were carried out on a Jasco FTIR - 6000 series spectrometer. The protein sample spectra were collected with a liquid helium cooled bolometer in the 15–250 cm^−1^ spectral range. The 15–100 cm^−1^ THz spectra were collected with a 25-micron beam splitter while the data in the 100–250 cm^−1^ spectral region was collected with a 12-micron beam splitter. For each transmission measurement a 25 mm diameter region of the protein sample was illuminated with the THz beam to determine the absorbance. In the spectral measurements presented, each scan consists of 16 averaged scans and the infrared data was collected with a spectral resolution of 4 cm^−1^.

### Molecular Dynamic Simulations

#### MD simulation of dark state rhodopsin and Meta II

Each MD simulation consisted of a starting x-ray crystal structure taken from the PDB database. PDB structure 1u19 was used for the inactive (dark) state of rhodopsin and 3pxo was used for Meta II. In all simulations, the receptor was embedded in a hydrated lipid bilayer with all atoms represented explicitly. Specifically, the dark-state receptor and any resolved water molecules from the crystal structure were embedded in an equilibrated palmitoyloleoyl-phosphatidylcholine (POPC) bilayer consisting of 110 lipid molecules, and additional 7400 water molecules, and 100 mM NaCl (to neutralize the net charge of the system). The membrane system was built with the use of the g_membed tool in Gromacs. All titratable groups in the receptor were considered to be charged^81^. The exceptions were Asp83 and Glu122, which were both neutral in both the dark-state and Meta II MD simulations. Also for the dark-state MD simulation, the Schiff base was protonated whereas Glu113 was deprotonated. For the Meta II simulation both the Schiff base and Glu113 were set to neutral. The active state receptor combined with the structural waters from the crystal structure was prepared in a similar manner to that of the dark-state. MD simulations were performed at 300 K using the Gromacs package (www.gromacs.org) version 5.0. The GROMOS96 43a2 force field parameters were utilized for the protein and the Berger lipid parameters were used for the lipid component of the membrane protein^82^. The SPC water model was used for hydration and the ground-state retinal parameters^83^ for both the 11-*cis* and *all-trans* retinal chromophore were obtained from the Bondar group.

In the rhodopsin simulations, energy minimizations were initially carried out to reduce the number of unfavorable contacts between added solvent molecules and the receptor using a steepest descent method to a convergence tolerance of 0.001 kJ mol^−1^. The energy minimization was followed by a MD run with constraints for 200 ps in which an isotropic force constant of 100 kJ mol^−1^ nm^−1^ was used on the protein and lipid atoms. During the restrained dynamics simulation, the temperature and pressure of the system were kept constant by weak coupling to a modified velocity rescaled Berendsen temperature^84^ and pressure baths and in all cases the protein, lipid, water, and ions were coupled to the temperature and pressure baths separately. The output conformation from the MD simulation with constraints was used as the starting conformation for an initial 200 ns equilibrium MD simulation.

Six subsequent simulations were conducted where randomized conformations from the last 10 ns of the equilibrium simulations were used as starting point conformations for each distinct simulation. These subsequent simulations were carried out for an additional 600 ns and were eventually used to assess the picosecond time scale fluctuations in the receptor systems. The final simulations were carried out with a 1 fs time step where the bonds between the hydrogen and the other heavier atoms were restrained to their equilibrium values with the linear constraints (LINCS) algorithm^85^. Particle mesh Ewald (PME) method^86^ was used to calculate the long-range electrostatic interactions in the simulation and was used with a real-space cutoff of 1.0 nm, a fourth order B-spline interpolation and a minimum grid spacing of 0.14 nm.

Trajectory snapshots, each containing a record of the atom positions and velocities at a particular instant in time, were saved every 100 fs during the production simulations.

#### MD simulation of rhodopsin bound to arrestin

MD simulations were also performed for a constitutively active form of human rhodopsin bound to arrestin (pdb 4zwj). For the rhodopsin-arrestin complex, the arrestin from the original crystal structure was kept in the same proximity to the receptor for the MD simulations carried out in this work but the T4-lysozyme was removed from the N-terminus. Missing residues in arrestin were evaluated with the homology modeling software program MODELLER (https://salilab.org/modeller/) to assess and reconstruct the missing regions in the three-dimensional structure. A “Slow” folding assignment method was used and a conformation with the lowest Discrete Optimized Protein Energy (DOPE) score was chosen for arrestin construction. Using the Meta II structure from a previous MD simulation as a model, we also added an *all-trans* retinal agonist to the receptor structure. The receptor, arrestin, (retinal) ligand, and resolved structural water molecules from the crystal structure were all embedded into a fully hydrated (POPC) lipid bilayer where all atoms were represented explicitly. The total system was comprised of approximately 65,000 atoms that included the rhodopsin-arrestin complex, 152 lipid molecules, 16,618 water molecules, and 100 mM NaCl to neutralize the net charge of the system. The MD simulation set-up and production runs were carried out in a similar manner as outlined for Meta II rhodopsin described in the previous section. Although, due to the large number of atoms in the rhodopsin-arrestin complex the initial run was conducted for 100 ns and the production MD simulation runs consisted of five independent simulations that were carried out for 300 ns.

## Calculations

### Velocity autocorrelation function

The velocity autocorrelation function (VACF) of atoms from the MD simulations were computed with the extended analysis tools that are included as part of the Gromacs software package. The VACF is defined by


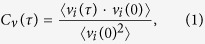


where *v* refers to velocity and *i* denotes an atom or molecule in the simulation system. Fourier Transform of the VACF (VACFFT) is used to project out the underlying frequencies of the molecular fluctuations associated with the correlated motions detected in the MD simulation.

It is important to note that the calculated fluctuations uncovered from the MD simulation are not always IR active but often coincides with the IR modes detected in the experimental investigation. However, we have also observed that the vibrational frequencies calculated in the MD simulations are often 10–15 cm^−1^ blue-shifted when compared with the experimentally observed IR modes. This is particularly the case in the global fluctuation region (≤100 cm^−1^) where the collective protein fluctuations have greater anharmonicity.

### Hydrogen-bond (H-bond) fluctuations and electrostatic interactions

Dynamical fluctuations within the hydrogen bond network within the receptor or between the receptor and the ligand and/or solvent molecules have been characterized by use of a correlation function


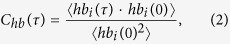


which averages over hydrogen bond pairs, and has an either 0 or 1 (hb (τ) = [0,1]) for a particular hydrogen bond *i* at time *t*. In this analysis, a hydrogen bond is defined by using a geometrical criterion, where the center of mass distance is less than 3.5 Å, the r(O•••H) distance is smaller than 2.6 Å, and the ∠HO•••O angle is smaller than 30°. Other weak interactions (Van der waal, electrostatic, etc…) in the receptor system are identified as contacts within an appropriate cut-off distance. We have used analyses of the weak interactions from the MD simulations as a means to further investigate the role and origin of ligand-receptor coupling in rhodopsin. We monitored the retinal torsional fluctuations as a function of residue-ligand interactions in the receptor ligand-binding pocket and used this information to further explore how such interactions are (allosterically) coupled to the collective fluctuations taking place within the receptor. This was primarily assessed by observing how correlated local (structural) fluctuations originating from the receptor ligand-binding pocket were coupled to the global motions of the receptor.

### Principal component analysis (PCA)

Principal component analyses (PCAs) were carried out by diagonalzing the covariance matrix 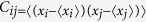, where *x* denotes protein atomic positions in the 3*N*-dimensional conformational space and the angular brackets represent the averages over the MD trajectory. Translational and rotational motions were removed by a least squares fitting to a reference structure. The eigenvectors of ***C*** were determined by diagonalization with an orthonormal transformation matrix. The resulting eigenvectors from the transformation were used to determine the PCA modes with eigenvalues (*λ*) equivalent to the variance in the direction of the corresponding eigenvector. The MD trajectory was projected onto the principal modes to determine the principal components. The eigenvalues *λ*_*i*_ of the principal components denote the mean square fluctuation of the principal component *i* and are arranged so that 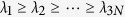. Using this arrangement, the trajectories were filtered along the first principal component to analyze the collective dynamics taking place within the protein. The cosine content of the PCA modes presented were found to be less than 0.001.

### Localized and global structural fluctuations (LSFs and GSFs)

The localized structural fluctuations (LSFs) were calculated with the method of Pandini *et al*.^87^ that utilizes a structural alphabet (SA) to define protein local structural fluctuations that are described by a set of 25 canonical states composed of four-residue protein fragments. Briefly, the four-residue fragments define the most probable protein local, conformational fluctuations in the protein 3-D structure. Structural correlations between local conformational changes of two protein fragments were calculated as a positional mutual information (MI) matrix between two column positions in the SA alignment. Likewise, the correlation between the motion of a protein fragment and the collective fluctuations (global structural fluctuations or GSFs) were calculated from the normalized MI of an array of fragment states and the array of collective states that were obtained from the top two PCAs of the receptor in the distinct states. This allowed us to investigate the relationship (overlap) between the local conformational fluctuations taking place in the receptor and the global, collective motions. In all cases, the outputs of the MI matrices were visualized with the use of network models.

### Multiple sequence alignment (MSA), conservation, and coevolution analysis

The Class A Rhodopsin-like MSA data set was retrieved from the GPCR database (http://gpcrdb.org/). The reference sequence and structure were set as opsd_bovin with the PDB code 1u19. The conservation and co-evolutionary analyses on the rhodopsin-like family of sequences were carried out with the MISTIC server (http://mistic.leloir.org.ar/index.php). The mutual information score as implemented in MISTIC is calculated between pairs of columns in the MSA. The frequency for each amino acid pair is calculated using sequence weighting along with low count corrections and compared with the expected frequency. It is assumed that mutations between amino acids are uncorrelated. The MI score is calculated as a weighted sum of the log ratios between the observed and expected amino acid pair frequencies. The MI scores were translated into MI *z*-scores by comparing the MI values for each pair of positions with a distribution of prediction scores obtained from a large set of randomized MSAs^88^. The *z*-score is then calculated as the number of standard deviations that the observed MI value falls above the mean value obtained from the randomized MSAs. A *z*-score threshold of 6.5 describes a sensitivity of 0.4 and a specificity of 0.95. MISTIC lists every MI value between two residues with a value ≥6.5.

### Determination of hinge residues in rhodopsin

The hinge residues in rhodopsin were identified with the webserver HingeProt (http://bioinfo3d.cs.tau.ac.il/HingeProt/hingeprot.html).

### Visualization of networks

Only the top 500 MI network links and nodes from the MSA were visualized. The position of residues in the two-dimensional MSA networks was computed with a combination of classical scaling and stress minimization^89^. And the network groupings were based on community detection resulting from modularity maximization^90^. The network layout and grouping were calculated using the software tool Visone (http://visone.info/).

## Additional Information

**How to cite this article**: Woods, K. N. *et al*. Vibrational resonance, allostery, and activation in rhodopsin-like G protein-coupled receptors. *Sci. Rep.* **6**, 37290; doi: 10.1038/srep37290 (2016).

**Publisher’s note**: Springer Nature remains neutral with regard to jurisdictional claims in published maps and institutional affiliations.

## Supplementary Material

Supplementary Information

## Figures and Tables

**Table 1:** Tentative spectral assignment of *collective fluctuations* (THz spectral range ≤100 cm^−1^) based on MD simulations.

Description from MD simulations	Assignment from experimental THz spectra
R-P(10-L): Collective oscillation of (polyene) chain terminus coupled with active-state receptor global mode	
R-P(20-L): Collective chain-twisting torsion coupled with global dynamics of the active-state receptor	
P(30-DL): Global oscillation of receptor side-chains coupled with an out-of-plane collective backbone torsion	(25-D): Global oscillation of receptor side-chains
R-P(40-DL): Chain torsional fluctuation in which the β-ionone ring motion is restricted by ligand pocket residue interactions	
R-P(65-D): Chain torsion combined with a retinal ring bending motion that is coupled with a collective receptor backbone torsion	(55-D): Collective backbone torsional oscillation
R-P(85-D): Collective oscillation of receptor amino acid side-chains coupled with the torsional fluctuation of the terminal portion of the retinal polyene chain	(75-D): Receptor residue-ligand coupled fluctuation involving retinal polyene chain

D, THz spectra in dark or 11-*cis* retinal (cm^−1^) in MD simulation; L, light or *all-trans* retinal (cm^−1^) in MD simulation, R = retinal, P = protein, and R-P = retinal-protein. All numbers are given as approximate cm^−1^ values for simulated MD (column 1) or experimental spectra (column 2).

**Table 2:** Tentative spectral assignment of more *localized fluctuations* (THz spectral range >100 cm^−1^) based on MD simulations.

Description from MD simulations	Assignment from experimental THz spectra
P(110-DL): Rotational fluctuation of methyl group side-chains	(110-L): Torsional oscillation of methyl groups in solvent-exposed helical regions
R-P(120-L): Chain torsion involving C9-and C13- methyl group fluctuations coupled with solvent-induced receptor helical fluctuations	122-L): Solvent-induced (anharmonic) H-bond fluctuation coupling solvent modes to receptor helical backbone atoms
R-P(130-L): Chain torsion involving C13- methyl group fluctuation coupled with solvent-induced receptor helical fluctuations	(128-L): Solvent-induced (anharmonic) H-bond fluctuation coupling solvent modes to receptor helical mainchain atoms
R-P(150-DL): Torsional oscillation of β-ionone ring joined with a localized fluctuation of retinal chain that couple with solvent-induced receptor helical fluctuations	(142-DL): Solvent-induced H-bond helical fluctuation coupled with out-of-plane retinal torsion
R-P(160-DL): Localized fluctuation of the retinal chain coupled with receptor interhelical H-bond fluctuations	(150-DL): Interhelical H-bond fluctuation coupled with out-of-plane retinal torsion

D, THz spectra in dark or 11-*cis* retinal (cm^−1^) in MD simulation; L, light or *all-trans* retinal (cm^−1^) in MD simulation, R = retinal, P = protein, and R-P = retinal-protein. All numbers are given as approximate cm^−1^ values for simulated MD (column 1) or experimental spectra (column 2).

## References

[b1] Perez D. M. & Karnik S. S. Multiple Signaling States of G-Protein-Coupled Receptors. Pharmacol. Rev. 57, 147–161 (2005). 15914464 10.1124/pr.57.2.2

[b2] Marinissen M. J. & Gutkind J. S. G-protein-coupled receptors and signaling networks: emerging paradigms. Trends Pharmacol. Sci. 22, 368–376 (2001). 11431032 10.1016/s0165-6147(00)01678-3

[b3] Benovic J. L. G-protein-coupled receptors signal victory. Cell 151, 1148–1150 (2012). 23201142 10.1016/j.cell.2012.11.015

[b4] Ghosh E., Kumari P., Jaiman D. & Shukla A. K. Methodological advances: the unsung heroes of the GPCR structural revolution. Nat. Rev. Mol. Cell Biol. 16, 69–81 (2015). 25589408 10.1038/nrm3933

[b5] Doré A. S. . Structure of class C GPCR metabotropic glutamate receptor 5 transmembrane domain. Nature 511, 557–562 (2014). 25042998 10.1038/nature13396

[b6] Park S. H. . Structure of the chemokine receptor CXCR1 in phospholipid bilayers. Nature 491, 779–783 (2012). 23086146 10.1038/nature11580 PMC3700570

[b7] Rasmussen S. G. F. . Crystal structure of the β2 adrenergic receptor-Gs protein complex. Nature 477, 549–555 (2011). 21772288 10.1038/nature10361 PMC3184188

[b8] Zhang C. . High-resolution crystal structure of human protease-activated receptor 1. Nature 492, 387–392 (2012). 23222541 10.1038/nature11701 PMC3531875

[b9] Wolf S. & Grünewald S. Sequence, Structure and Ligand Binding Evolution of Rhodopsin-Like G Protein-Coupled Receptors: A Crystal Structure-Based Phylogenetic Analysis. PLOS ONE 10, e0123533 (2015). 25881057 10.1371/journal.pone.0123533 PMC4399913

[b10] Klein-Seetharaman J. . Differential dynamics in the G protein-coupled receptor rhodopsin revealed by solution NMR. Proc. Natl. Acad. Sci. USA 101, 3409–3413 (2004). 14990789 10.1073/pnas.0308713101 PMC373475

[b11] Klein-Seetharaman J., Getmanova E. V., Loewen M. C., Reeves P. J. & Khorana H. G. NMR spectroscopy in studies of light-induced structural changes in mammalian rhodopsin: Applicability of solution 19F NMR. Proc. Natl. Acad. Sci. USA 96, 13744–13749 (1999). 10570143 10.1073/pnas.96.24.13744 PMC24135

[b12] Liu J. J., Horst R., Katritch V., Stevens R. C. & Wüthrich K. Biased signaling pathways in β2-adrenergic receptor characterized by 19F-NMR. Science 335, 1106–1110 (2012). 22267580 10.1126/science.1215802 PMC3292700

[b13] Christopoulos A. Allosteric binding sites on cell-surface receptors: novel targets for drug discovery. Nat. Rev. Drug Discov. 1, 198–210 (2002). 12120504 10.1038/nrd746

[b14] Conn P. J., Christopoulos A. & Lindsley C. W. Allosteric modulators of GPCRs: a novel approach for the treatment of CNS disorders. Nat. Rev. Drug Discov. 8, 41–54 (2009). 19116626 10.1038/nrd2760 PMC2907734

[b15] Flock T. . Universal allosteric mechanism for Gα activation by GPCRs. Nature 524, 173–179 (2015). 26147082 10.1038/nature14663 PMC4866443

[b16] Standfuss J. . Crystal structure of constitutively active rhodopsin: How an agonist can activate its GPCR. Nature 471, 656–660 (2011). 21389983 10.1038/nature09795 PMC3715716

[b17] Scheerer P. . Crystal structure of opsin in its G-protein-interacting conformation. Nature 455, 497–502 (2008). 18818650 10.1038/nature07330

[b18] Deupi X. . Stabilized G protein binding site in the structure of constitutively active metarhodopsin-II. Proc. Natl. Acad. Sci. 109, 119–124 (2012). 22198838 10.1073/pnas.1114089108 PMC3252945

[b19] Kang Y. . Crystal structure of rhodopsin bound to arrestin by femtosecond X-ray laser. Nature 523, 561–567 (2015). 26200343 10.1038/nature14656 PMC4521999

[b20] Daily M. D. & Gray J. J. Local motions in a benchmark of allosteric proteins. Proteins Struct. Funct. Bioinforma. 67, 385–399 (2007). 10.1002/prot.21300 17295319

[b21] Pandini A., Fornili A., Fraternali F. & Kleinjung J. Detection of allosteric signal transmission by information-theoretic analysis of protein dynamics. FASEB J 26, 868–881 (2012). 22071506 10.1096/fj.11-190868 PMC3290435

[b22] Unal H. & Karnik S. S. Domain coupling in GPCRs: the engine for induced conformational changes. Trends Pharmacol. Sci. 33, 79–88 (2012). 22037017 10.1016/j.tips.2011.09.007 PMC3273637

[b23] Leioatts N., Romo T. D., Danial S. A. & Grossfield A. Retinal Conformation Changes Rhodopsin’s Dynamic Ensemble. Biophys. J 109, 608–617 (2015). 26244742 10.1016/j.bpj.2015.06.046 PMC4572577

[b24] Lau P.-W., Grossfield A., Feller S. E., Pitman M. C. & Brown M. F. Dynamic structure of retinylidene ligand of rhodopsin probed by molecular simulations. J. Mol. Biol. 372, 906–917 (2007). 17719606 10.1016/j.jmb.2007.06.047 PMC5233727

[b25] Isin B., Rader A., Dhiman H., Klein-Seetharaman J. & Bahar I. Predisposition of the dark state of rhodopsin to functional changes in structure. Proteins 65, 970–983 17009319 10.1002/prot.21158

[b26] Kahsai A. W. . Multiple ligand-specific conformations of the β2-adrenergic receptor. Nat. Chem. Biol. 7, 692–700 (2011). 21857662 10.1038/nchembio.634 PMC3404607

[b27] Kenakin T. Efficacy at g-protein-coupled receptors. Nat. Rev. Drug Discov. 1, 103–110 (2002). 12120091 10.1038/nrd722

[b28] Kenakin T. Functional selectivity and biased receptor signaling. J. Pharmacol. Exp. Ther. 336, 296–302 (2011). 21030484 10.1124/jpet.110.173948

[b29] Deupi X. & Kobilka B. K. Energy Landscapes as a Tool to Integrate GPCR Structure, Dynamics, and Function. Physiology 25, 293–303 (2010). 20940434 10.1152/physiol.00002.2010 PMC3056154

[b30] Niesen M. J. M., Bhattacharya S. & Vaidehi N. The Role of Conformational Ensembles in Ligand Recognition in G-Protein Coupled Receptors. J. Am. Chem. Soc. 133, 13197–13204 (2011). 21766860 10.1021/ja205313h

[b31] Vaidehi N. & Kenakin T. The role of conformational ensembles of seven transmembrane receptors in functional selectivity. Curr. Opin. Pharmacol. 10, 775–781 (2010). 20933468 10.1016/j.coph.2010.09.004

[b32] LeVine M. V. & Weinstein H. NbIT–a new information theory-based analysis of allosteric mechanisms reveals residues that underlie function in the leucine transporter LeuT. PLoS Comput. Biol. 10, e1003603 (2014). 24785005 10.1371/journal.pcbi.1003603 PMC4006702

[b33] Lamichhane R. . Single-molecule view of basal activity and activation mechanisms of the G protein-coupled receptor β2AR. Proc. Natl. Acad. Sci. USA 112, 14254–14259 (2015). 26578769 10.1073/pnas.1519626112 PMC4655547

[b34] Manglik A. . Structural Insights into the Dynamic Process of β2-Adrenergic Receptor Signaling. Cell 161, 1101–1111 (2015). 25981665 10.1016/j.cell.2015.04.043 PMC4441853

[b35] Bockenhauer S., Fürstenberg A., Yao X. J., Kobilka B. K. & Moerner W. E. Conformational dynamics of single G protein-coupled receptors in solution. J. Phys. Chem. B 115, 13328–13338 (2011). 21928818 10.1021/jp204843r PMC3213290

[b36] Frauenfelder H., Sligar S. & Wolynes P. The energy landscapes and motions of proteins. Science 254, 1598–1603 (1991). 1749933 10.1126/science.1749933

[b37] Bakan A. & Bahar I. The intrinsic dynamics of enzymes plays a dominant role in determining the structural changes induced upon inhibitor binding. Proc. Natl. Acad. Sci 106, 14349–14354 (2009). 19706521 10.1073/pnas.0904214106 PMC2728110

[b38] Barends T. R. M. . Direct observation of ultrafast collective motions in CO myoglobin upon ligand dissociation. Science 350, 445–450 (2015). 26359336 10.1126/science.aac5492

[b39] Kobilka B. K. & Deupi X. Conformational complexity of G-protein-coupled receptors. Trends Pharmacol. Sci. 28, 397–406 (2007). 17629961 10.1016/j.tips.2007.06.003

[b40] Daily M. D. & Gray J. J. Local motions in a benchmark of allosteric proteins. Proteins 67, 385–399 (2007). 17295319 10.1002/prot.21300

[b41] Deupi X. Relevance of rhodopsin studies for GPCR activation. Biochim. Biophys. Acta 1837, 674–682 (2014). 24041646 10.1016/j.bbabio.2013.09.002

[b42] Tsai C.-J. & Nussinov R. A Unified View of ‘How Allostery Works’. PLOS Comput Biol 10, e1003394 (2014). 24516370 10.1371/journal.pcbi.1003394 PMC3916236

[b43] Balabin I. A., Yang W. & Beratan D. N. Coarse-grained modeling of allosteric regulation in protein receptors. Proc. Natl. Acad. Sci. 106, 14253–14258 (2009). 19706508 10.1073/pnas.0901811106 PMC2732817

[b44] Palczewski K. . Crystal structure of rhodopsin: A G protein-coupled receptor. Science 289, 739–745 (2000). 10926528 10.1126/science.289.5480.739

[b45] Choe H.-W. . Crystal structure of metarhodopsin II. Nature 471, 651–655 (2011). 21389988 10.1038/nature09789

[b46] Brown M. F., Martínez-Mayorga K., Nakanishi K., Salgado G. F. J. & Struts A. V. Retinal Conformation and Dynamics in Activation of Rhodopsin Illuminated by Solid-state 2H NMR Spectroscopy. Photochem. Photobiol. 85, 442–453 (2009). 19267870 10.1111/j.1751-1097.2008.00510.x PMC2858981

[b47] Paciaroni A., Bizzarri A. R. & Cannistraro S. Neutron scattering evidence of a boson peak in protein hydration water. Phys. Rev. E Stat. Phys. Plasmas Fluids Relat. Interdiscip. Top. 60, R2476–R2479 (1999). 10.1103/physreve.60.r2476 11970180

[b48] Woods K. N. Solvent-induced backbone fluctuations and the collective librational dynamics of lysozyme studied by terahertz spectroscopy. Phys. Rev. E 81, 31915 (2010). 10.1103/PhysRevE.81.031915 20365778

[b49] Gervasio F. L., Cardini G., Salvi P. R. & Schettino V. Low-Frequency Vibrations of all-trans-Retinal: Far-Infrared and Raman Spectra and Density Functional Calculations. J. Phys. Chem. A 102, 2131–2136 (1998).

[b50] Struts A. V., Salgado G. F. J., Martínez-Mayorga K. & Brown M. F. Retinal dynamics underlie its switch from inverse agonist to agonist during rhodopsin activation. Nat. Struct. Mol. Biol. 18, 392–394 (2011). 21278756 10.1038/nsmb.1982 PMC5283944

[b51] Leioatts N. . Retinal Ligand Mobility Explains Internal Hydration and Reconciles Active Rhodopsin Structures. Biochemistry (Mosc.) 53, 376–385 (2014). 10.1021/bi4013947 PMC4096112 24328554

[b52] Woods K. N. THz time scale structural rearrangements and binding modes in lysozyme-ligand interactions. J. Biol. Phys. 40, 121–137 (2014). 24682643 10.1007/s10867-014-9341-4 PMC4049381

[b53] Heyden M. . Dissecting the THz spectrum of liquid water from first principles via correlations in time and space. Proc. Natl. Acad. Sci. 107, 12068–12073 (2010). 20566886 10.1073/pnas.0914885107 PMC2901429

[b54] Woods K. N. The glassy state of crambin and the THz time scale protein-solvent fluctuations possibly related to protein function. BMC Biophys. 7, 8 (2014). 25184036 10.1186/s13628-014-0008-0 PMC4143578

[b55] Woods K. N. & Pfeffer J. Using THz spectroscopy, evolutionary network analysis methods, and MD simulation to map the evolution of allosteric communication pathways in c-type lysozymes. Mol. Biol. Evol., doi: 10.1093/molbev/msv178 msv178 (2015). PMC4693973 26337549

[b56] Woods K. N. Using THz time-scale infrared spectroscopy to examine the role of collective, thermal fluctuations in the formation of myoglobin allosteric communication pathways and ligand specificity. Soft Matter 10, 4387–4402 (2014). 24801988 10.1039/c3sm53229a

[b57] Okada T. . Functional role of internal water molecules in rhodopsin revealed by x-ray crystallography. Proc. Natl. Acad. Sci. USA 99, 5982–5987 (2002). 11972040 10.1073/pnas.082666399 PMC122888

[b58] Angel T. E., Chance M. R. & Palczewski K. Conserved waters mediate structural and functional activation of family A (rhodopsin-like) G protein-coupled receptors. Proc. Natl. Acad. Sci. 106, 8555–8560 (2009). 19433801 10.1073/pnas.0903545106 PMC2688986

[b59] Ballesteros J. A. & Weinstein H. In Methods in Neurosciences (ed. Sealfon S. C.) 25, 366–428 (Academic Press, 1995).

[b60] Hofmann K. P. . A G protein-coupled receptor at work: the rhodopsin model. Trends Biochem. Sci. 34, 540–552 (2009). 19836958 10.1016/j.tibs.2009.07.005

[b61] Trzaskowski B. . Action of Molecular Switches in GPCRs - Theoretical and Experimental Studies. Curr. Med. Chem. 19, 1090–1109 (2012). 22300046 10.2174/092986712799320556 PMC3343417

[b62] Boehr D. D., Nussinov R. & Wright P. E. The role of dynamic conformational ensembles in biomolecular recognition. Nat. Chem. Biol. 5, 789–796 (2009). 19841628 10.1038/nchembio.232 PMC2916928

[b63] Giraud G., Karolin J. & Wynne K. Low-Frequency Modes of Peptides and Globular Proteins in Solution Observed by Ultrafast OHD-RIKES Spectroscopy. Biophys. J 85, 1903–1913 (2003). 12944303 10.1016/S0006-3495(03)74618-9 PMC1303362

[b64] Conti Nibali V., D’Angelo G., Paciaroni A., Tobias D. J. & Tarek M. On the Coupling between the Collective Dynamics of Proteins and Their Hydration Water. J. Phys. Chem. Lett. 5, 1181–1186 (2014). 26274468 10.1021/jz500023e

[b65] Fritze O. . Role of the conserved NPxxY(x)5,6F motif in the rhodopsin ground state and during activation. Proc. Natl. Acad. Sci. USA 100, 2290–2295 (2003). 12601165 10.1073/pnas.0435715100 PMC151333

[b66] Krishnan A., Almén M. S., Fredriksson R. & Schiöth H. B. The Origin of GPCRs: Identification of Mammalian like Rhodopsin, Adhesion, Glutamate and Frizzled GPCRs in Fungi. PLoS ONE 7, e29817 (2012). 22238661 10.1371/journal.pone.0029817 PMC3251606

[b67] Süel G. M., Lockless S. W., Wall M. A. & Ranganathan R. Evolutionarily conserved networks of residues mediate allosteric communication in proteins. Nat. Struct. Mol. Biol. 10, 59–69 (2003). 10.1038/nsb881 12483203

[b68] Zaitseva E., Brown M. F. & Vogel R. Sequential Rearrangement of Interhelical Networks Upon Rhodopsin Activation in Membranes: The Meta IIa Conformational Substate. J. Am. Chem. Soc. 132, 4815–4821 (2010). 20230054 10.1021/ja910317a PMC2859452

[b69] Sakmar T. P., Menon S. T., Marin E. P. & Awad E. S. Rhodopsin: insights from recent structural studies. Annu. Rev. Biophys. Biomol. Struct. 31, 443–484 (2002). 11988478 10.1146/annurev.biophys.31.082901.134348

[b70] Liu Y. & Bahar I. Sequence Evolution Correlates with Structural Dynamics. Mol. Biol. Evol. 29, 2253–2263 (2012). 22427707 10.1093/molbev/mss097 PMC3424413

[b71] del Sol A., Fujihashi H., Amoros D. & Nussinov R. Residues crucial for maintaining short paths in network communication mediate signaling in proteins. Mol. Syst. Biol. 2, 2006.0019 (2006). 10.1038/msb4100063 PMC1681495 16738564

[b72] Kinoshita M. & Okada T. Structural conservation among the rhodopsin-like and other G protein-coupled receptors. Sci. Rep. 5, 9176 (2015). 25775952 10.1038/srep09176 PMC4361874

[b73] Nygaard R., Valentin-Hansen L., Mokrosinski J., Frimurer T. M. & Schwartz T. W. Conserved water-mediated hydrogen bond network between TM-I, -II, -VI, and -VII in 7TM receptor activation. J. Biol. Chem. 285, 19625–19636 (2010). 20395291 10.1074/jbc.M110.106021 PMC2885241

[b74] Hawkins R. J. & McLeish T. C. B. Coupling of Global and Local Vibrational Modes in Dynamic Allostery of Proteins. Biophys. J 91, 2055–2062 (2006). 16798805 10.1529/biophysj.106.082180 PMC1557547

[b75] Balu R. . Terahertz Spectroscopy of Bacteriorhodopsin and Rhodopsin: Similarities and Differences. Biophys. J 94, 3217–3226 (2008). 18199669 10.1529/biophysj.107.105163 PMC2275673

[b76] Kusnetzow A. K., Altenbach C. & Hubbell W. L. Conformational States and Dynamics of Rhodopsin in Micelles and Bilayers. Biochemistry (Mosc.) 45, 5538–5550 (2006). 10.1021/bi060101v PMC2739654 16634635

[b77] Oprian D. D., Molday R. S., Kaufman R. J. & Khorana H. G. Expression of a synthetic bovine rhodopsin gene in monkey kidney cells. Proc. Natl. Acad. Sci. USA 84, 8874–8878 (1987). 2962193 10.1073/pnas.84.24.8874 PMC299653

[b78] Reeves P. J., Kim J.-M. & Khorana H. G. Structure and function in rhodopsin: a tetracycline-inducible system in stable mammalian cell lines for high-level expression of opsin mutants. Proc. Natl. Acad. Sci. USA 99, 13413–13418 (2002). 12370422 10.1073/pnas.212519199 PMC129687

[b79] Hwa J., Reeves P. J., Klein-Seetharaman J., Davidson F. & Khorana H. G. Structure and function in rhodopsin: Further elucidation of the role of the intradiscal cysteines, Cys-110, -185, and −187, in rhodopsin folding and function. Proc. Natl. Acad. Sci. USA 96, 1932–1935 (1999). 10051572 10.1073/pnas.96.5.1932 PMC26714

[b80] Wexler A. & Hasegawa S. Relative Humidity-Temperature Relationships of Some Saturated Salt Solutions in the Temperature Range 0 to 50 C. J. Res. Natl. Bur. Stand. 53, 19–26 (1954).

[b81] Fahmy K. . Protonation states of membrane-embedded carboxylic acid groups in rhodopsin and metarhodopsin II: a Fourier-transform infrared spectroscopy study of site-directed mutants. Proc. Natl. Acad. Sci. USA 90, 10206–10210 (1993). 7901852 10.1073/pnas.90.21.10206 PMC47743

[b82] Berger O., Edholm O. & Jähnig F. Molecular dynamics simulations of a fluid bilayer of dipalmitoylphosphatidylcholine at full hydration, constant pressure, and constant temperature. Biophys. J 72, 2002–2013 (1997). 9129804 10.1016/S0006-3495(97)78845-3 PMC1184396

[b83] Bondar A.-N., Knapp-Mohammady M., Suhai S., Fischer S. & Smith J. Ground-State Properties of the Retinal Molecule: from Quantum Mechanical to Classical Mechanical Computations of Retinal Proteins. Theor. Chem. Acc. Theory Comput. Model. Theor. Chim. Acta 130, 1169–1183 (2011).

[b84] Berendsen H. J. C., Postma J. P. M., van Gunsteren W. F., DiNola A. & Haak J. R. Molecular dynamics with coupling to an external bath. J. Chem. Phys. 81, 3684 (1984).

[b85] Hess B., Bekker H., Berendsen H. J. C. & Fraaije J. G. E. M. LINCS: A linear constraint solver for molecular simulations. J. Comput. Chem. 18, 1463–1472 (1997).

[b86] Essmann U. . A smooth particle mesh Ewald method. J. Chem. Phys. 103, 8577–8593 (1995).

[b87] Pandini A., Fornili A., Fraternali F. & Kleinjung J. GSATools: analysis of allosteric communication and functional local motions using a structural alphabet. Bioinformatics 29, 2053–2055 (2013). 23740748 10.1093/bioinformatics/btt326 PMC3722520

[b88] Buslje C. M., Santos J., Delfino J. M. & Nielsen M. Correction for phylogeny, small number of observations and data redundancy improves the identification of coevolving amino acid pairs using mutual information. Bioinformatics 25, 1125–1131 (2009). 19276150 10.1093/bioinformatics/btp135 PMC2672635

[b89] Brandes U. & Pich C. An Experimental Study on Distance-Based Graph Drawing. in Graph Drawing (eds Tollis I. G. & Patrignani M.) 218–229 (Springer: Berlin Heidelberg,, 2008).

[b90] Newman M. E. J. Modularity and community structure in networks. Proc. Natl. Acad. Sci. 103, 8577–8582 (2006). 16723398 10.1073/pnas.0601602103 PMC1482622

