# Supplementary material for: Vibrational resonance, allostery, and activation in rhodopsin-like G protein-coupled receptors
Source: Sci Rep. 2016 Nov 16;6:37290. doi: 10.1038/srep37290 (PMC5110974; doi:10.1038/srep37290)
Supplement: Supplementary Information [file srep37290-s1.pdf]

Vibrational resonance, allostery, and activation in rhodopsin-like G protein-coupled receptors

Kristina N. Woods[1], Jürgen Pfeffer[2], Arpana Dutta[3], and Judith Klein-Seetharaman[3,4]
[1]*Physics Department, Carnegie Mellon University, Pittsburgh, PA 15213, USA*
[2]*Institute for Software Research, Carnegie Mellon University, Pittsburgh, PA 15213, USA*
[3]*Department of Structural Biology, University of Pittsburgh School of Medicine, Pittsburgh, PA 15260, USA*
[4] *Warwick Medical School, University of Warwick, Coventry CV4 7AL, UK*

Supplementary information

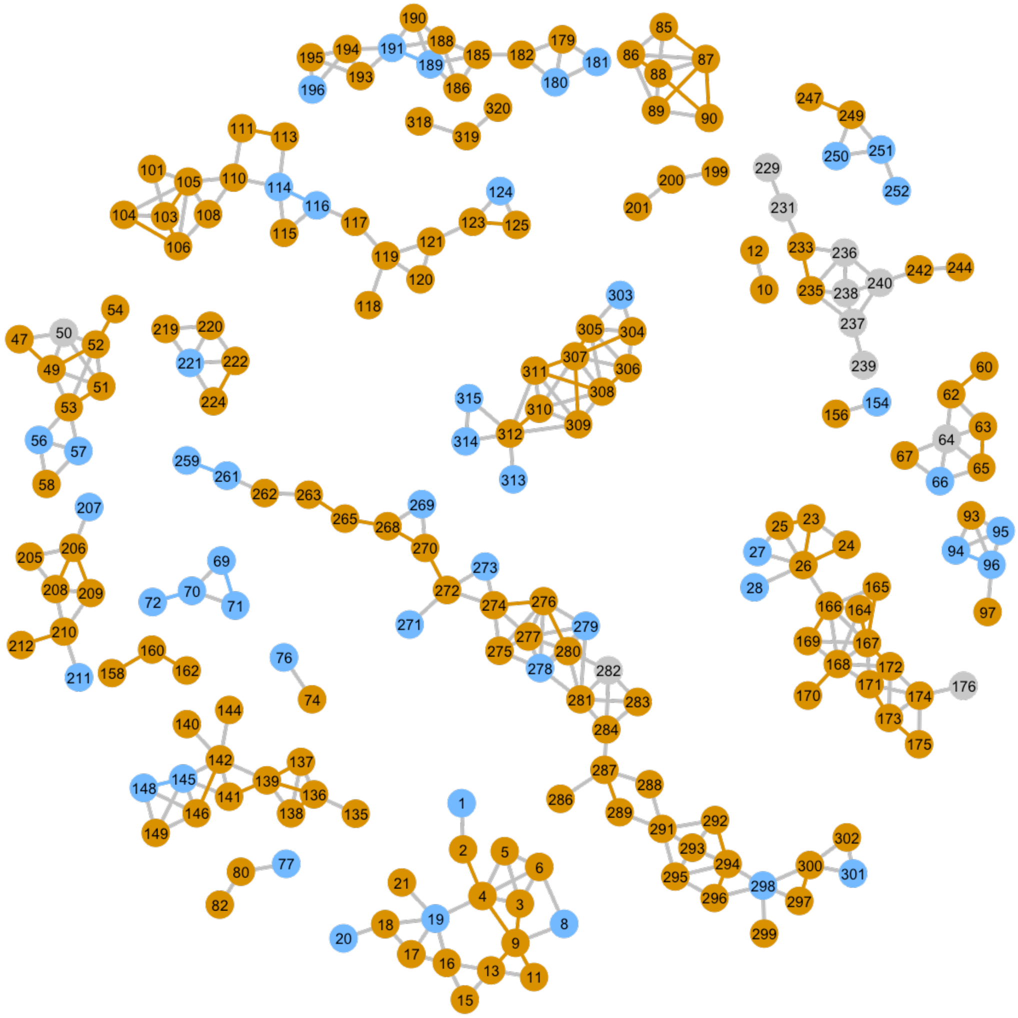

	Meta II	WT (inactive) Blue	WT (inactive) Blue in Meta II	WT (inactive) Orange	WT (inactive) Orange in Meta II
Number of Nodes	213	104	48 (46.2%)	225	154 (68.4%)
Number of Edges	324	558	7 (1.3%)	646	61 (9.4%)

Figure S1: Network representation the LSFs from the Meta-II rhodopsin MD simulation where the nodes are colored according to the separate network communities of the LSFs from inactive state rhodopsin in Figure 2. The gray nodes highlight residues that were not part of the LSF network of interactions in inactive rhodopsin.

Supplementary FDA: Changes in retinal-rhodopsin pairwise forces and the formation of allosteric communication pathways in rhodopsin (Figures S2 – S6)

In this investigation we have also extended the equilibrium MD simulations of rhodopsin in both the inactive and Meta II state to include force distribution analyses (FDA)[1]. The aim of these studies is to deduce the mechanical stress that is both distributed and propagated within the interior of the receptor due to the retinal interactions that take place within the ligand-binding pocket. And to further comprehend the influence of the retinal dynamics on the global modes of the receptor. A perturbation, such as ligand binding in the receptor structure, would be expected to lead to a shift in the distribution of conformational states across the ensemble. Thus, a return to equilibrium[2] requires a release of the strain energy through a series of propagating structural deformations. In general the fluctuations associated with the release of strain in proteins tend to be smaller-scale, conformational rearrangements rather than large-scale protein conformational changes. Moreover, the pathways for strain release[3] have been conjectured to be closely connected with the formation of major allosteric propagation pathways in proteins and enzymes. Therefore to grasp the nature of the network of correlated protein fluctuations that arise in response to the retinal in rhodopsin we have conducted PCA on the residue averaged pair-wise forces in in both the dark and active-state receptor. Hence, the interpretation of the propagation pathways uncovered from FDA relies on the observations from force-PCA analyses.

In Figs. S2-S3 we find that the retinal force on the receptor in the dark state and Meta II differs somewhat significantly. The force from the retinal in the dark-state is distributed more extensively on the receptor structure and also slightly more aggregated in the extracellular region when contrasted with Meta II. On the other hand, the Meta II receptor ligand-induced forces have less of an influence on the overall structure of the receptor yet the magnitude of the force-induced interactions is higher and concentrated primarily in the immediate vicinity of the retinal C9- and C13- methyl groups. From the MD simulations, we are able to establish a network of interactions that propagate internal strain from the ligand-binding site to the rest of the receptor structure via subtle, structural fluctuations. In the calculation of the pair-wise forces in the dark-state, we find that the retinal interaction with the receptor promotes two distinct mechanisms of force propagation that lead to conformational rearrangements within the receptor interior (Figs. S4a - c). The major pathway (PC1) in Fig. S4 involves a torsional compression of the retinal-ligand binding pocket that is primarily due to the force on residues Glu113 and Gly114 from the oscillation of the Schiff base linkage on helix 3 and from the force on Arg177 that mediates stability of the retinal in the dark-state of the receptor. The ligand- induced force on Glu113 and Gly114 is disseminated through the receptor as a torsional oscillation that promotes fluctuations of Gly89 – 90 on helix 2 and Gly120 on helix 3.

Analogously, the force on Arg177 induces oscillations in the β3 loop of extracellular loop 2 (EL2) that creates a counter-torque from that which is produced from the retinal force on residues 113 and 114. Hence, the propagation of internal stress reveals a network of correlated fluctuations that induces a receptor-wide torque-induced rotational motion that resembles the dominant PCA mode uncovered from the MD simulation of the dark-state receptor in Fig. S4d.

A minor force-induced propagation pathway (PC2) in the dark-state receptor is also uncovered in Fig. S4b. In this case, a transient fluctuation of the retinal intermittently modifies the ligand-receptor interaction such that the interaction with the C9- methyl group of the retinal has a much stronger interaction with Gly120 and Gly121. The fluctuation-induced modification in ligand-receptor interactions also creates a counter torque centered at Gly174. Together the retinal-induced correlated structural fluctuations are translated as an elongation motion that extends from the ligand-binding pocket into the direction of the G-protein binding, which consequently slightly alters the packing in the receptor hydrophobic core.

The retinal-induced forced in Meta II only reveals one major pathway of signal propagation (Fig. S5). The interaction with the retinal polyene tail induces prominent forces in residues lining the C9- methyl group, namely Leu119 – Glu122. The fluctuation-induced interaction also modifies the receptor interactions near the β-ionine ring. The oscillation of the ring makes close contact with Tyr268 inducing a counter torque centered at Gly270. Similar to the minor signal propagation pathway uncovered in the dark state receptor in Fig. S4b, the retinal-induced force creates an elongation torsion that extends in this case from the N-terminus up to the G-protein coupling region. Specifically, the torsional oscillation of residues 119 – 122 on helix 3 creates a correlated set a structural fluctuations that couples regions of the N-terminus, the CL2 loop between helices 3 and 4, and the C-terminus. The force-induced torsional oscillation centered at Gly270 modifies the dynamical fluctuations in intracellular regions of helix 5 as well as the CL3 loop separating helices 5 and 6. In this case (when contrasted with PC2 in the dark-state receptor) we also observe a more substantial altering of the hydrophobic packing in the receptor in the regions separating helices 3 and 6 that accompany the helical rearrangements. In fact we observe an overall "softening" of backbone motion in the global fluctuations of Meta II (Figs. S6a-b) that is due to the mechanical strain of force propagation from the retinal to the G-protein region that disrupts the packing of the receptor hydrophobic core and at the same time potentially enhances the affinity for the G-protein. The signal is carried via a network of correlated (in-plane) side-chain fluctuations that are distinct from those observed in the dark-state receptor (Fig. S6c) and the overall induced motion strongly resembles the dominant PCA mode uncovered from the MD simulation of Meta II (Fig. S5c).

It is interesting to note that experimentally we observe no prominent vibrational modes in the light-state receptor in the $\leq 100\ cm^{-1}$ region of the THz spectrum yet the MD simulation results suggest that there should be large-amplitude modes in that frequency region due to collective oscillations of both side-chain and backbone atoms (or a coupling of side-chain and backbone motions). On interesting thing to consider is that the strength of the infrared absorption is directly associated with the change in dipole moment. Restricted rotational oscillations or torsions create large changes in dipole moments; hence these types of motions are very prominent in the THz spectrum. For instance, we have associated the peaks at ~ 80 cm^{-1} and 65 cm^{-1} (Figs. 3a-b, Figs. S6a-b) with collective oscillations that are associated with a global hinge torsion that takes place in the dark-state of the receptor. These types of motions would be expected to prominent in the experimental spectrum and we find that they are (Fig. 3a). The in-plane fluctuations (between 30 – 40 cm^{-1}) arising from the retinal-induced structural fluctuations in Meta II (Fig. 3b and Figs. S6a-c) would

probably not create large changes in dipole moments. Hence, it is likely that their presence would not be dominant in the low- frequency region of the experimental spectrum. Although the computational analyses that we have conducted suggest that their role in the active receptor dynamics is significant.

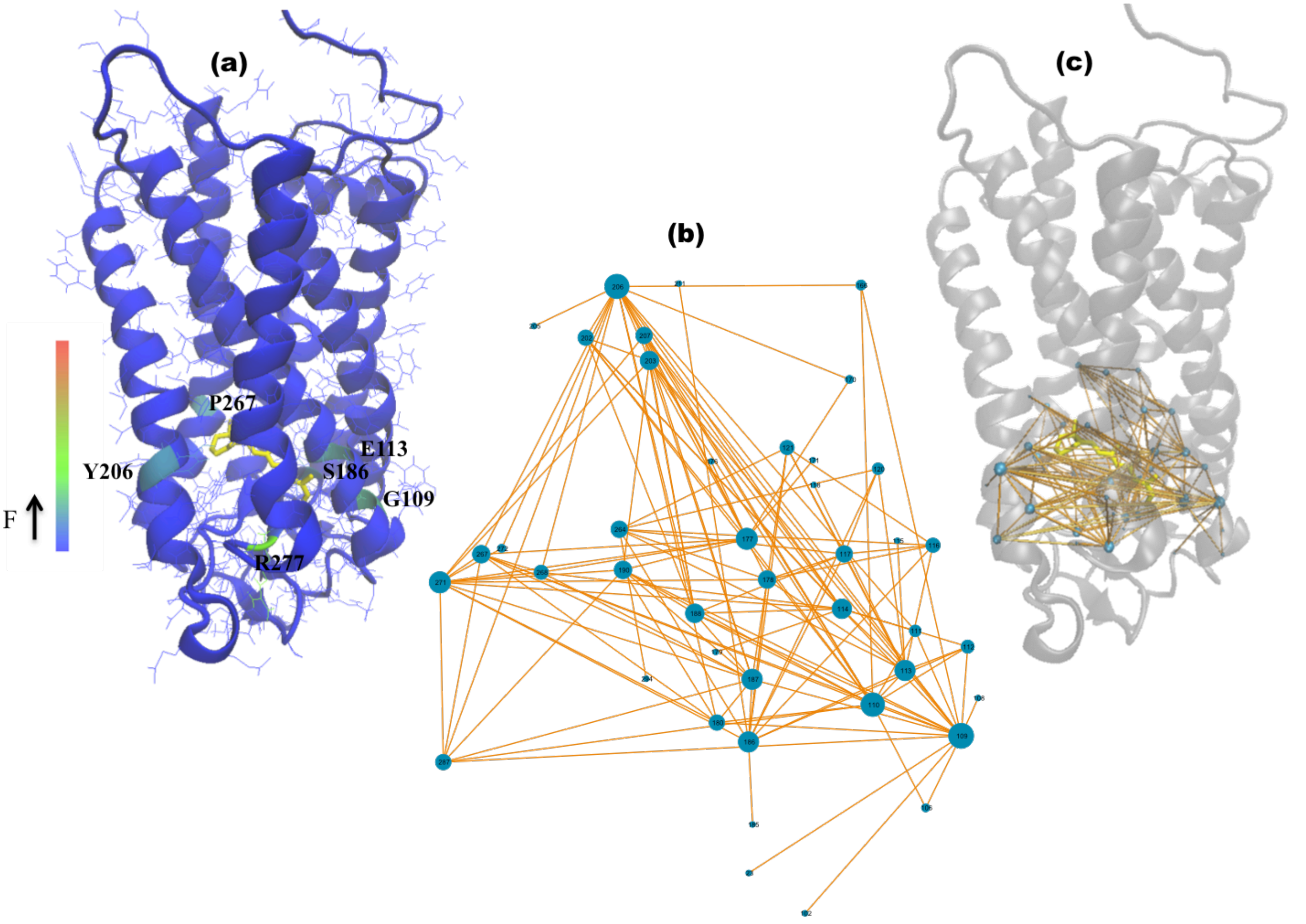

Figure S2: (a) Averaged pair-wise residue forces mapped onto a cartoon representation of the dark-state of rhodopsin. The color scheme ranges from blue to red, where blue describes regions with low residue force from interaction with the retinal and red high residue force. (b) Network representation of the pair-wise residue forces of dark-state rhodopsin. The nodes represent amino acid residues and the size of the nodes corresponds to the number of connections. The edges between the nodes represent pair-wise interactions. (c) The network representation of the pair-wise forces from (b) mapped onto a cartoon representation of rhodopsin.

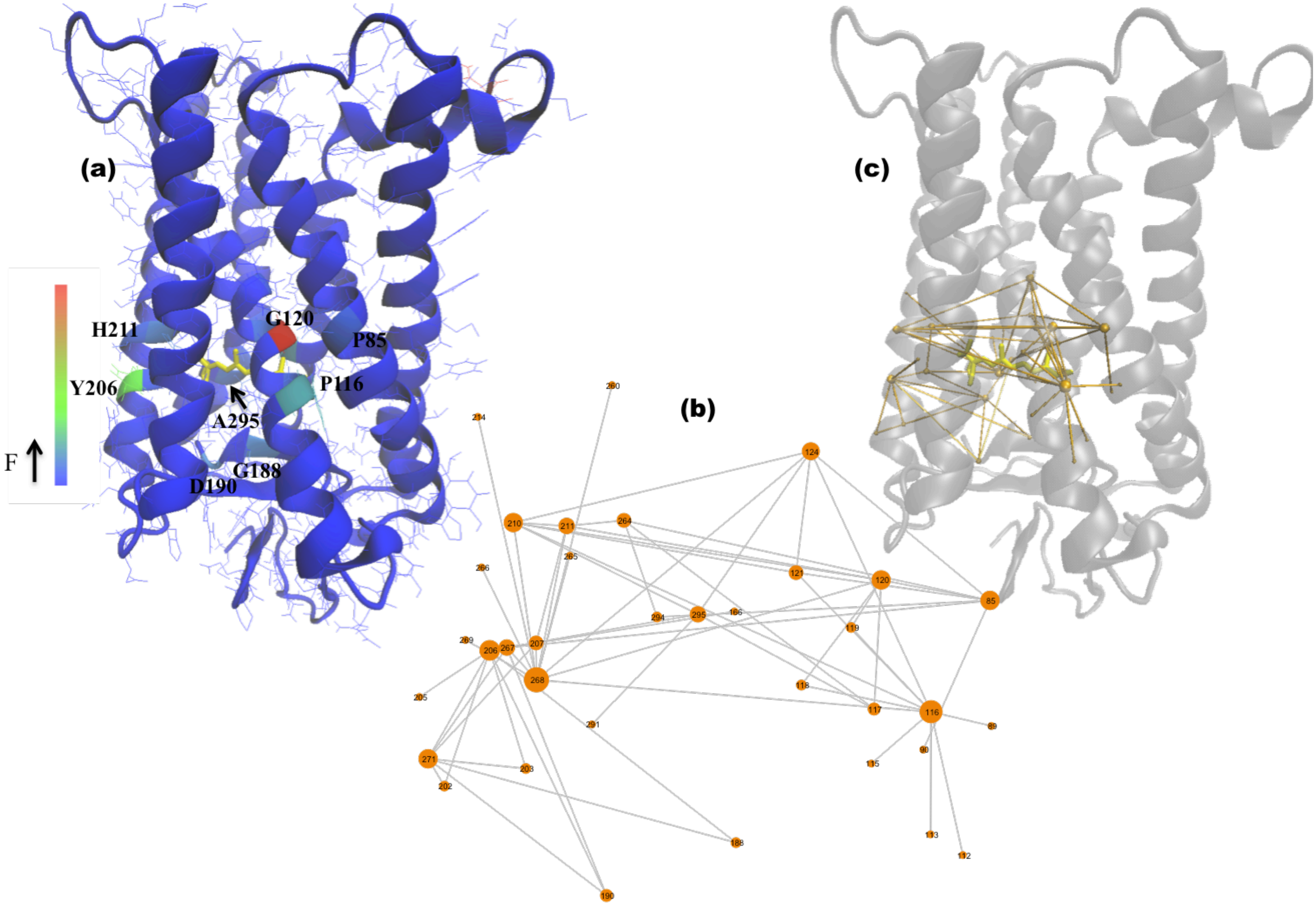

Figure S3: (a) Averaged pair-wise residue forces mapped onto a cartoon representation of Meta II rhodopsin. The color scheme ranges from blue to red, where blue describes regions with low residue force from interaction with the retinal and red high residue force. (b) Network representation of the pair-wise residue forces of Meta II. The nodes represent amino acid residues and the size of the nodes corresponds to the number of connections. The edges between the nodes represent pair-wise interactions. (c) The network representation of the pair-wise forces from (b) mapped onto a cartoon representation of Meta II rhodopsin.

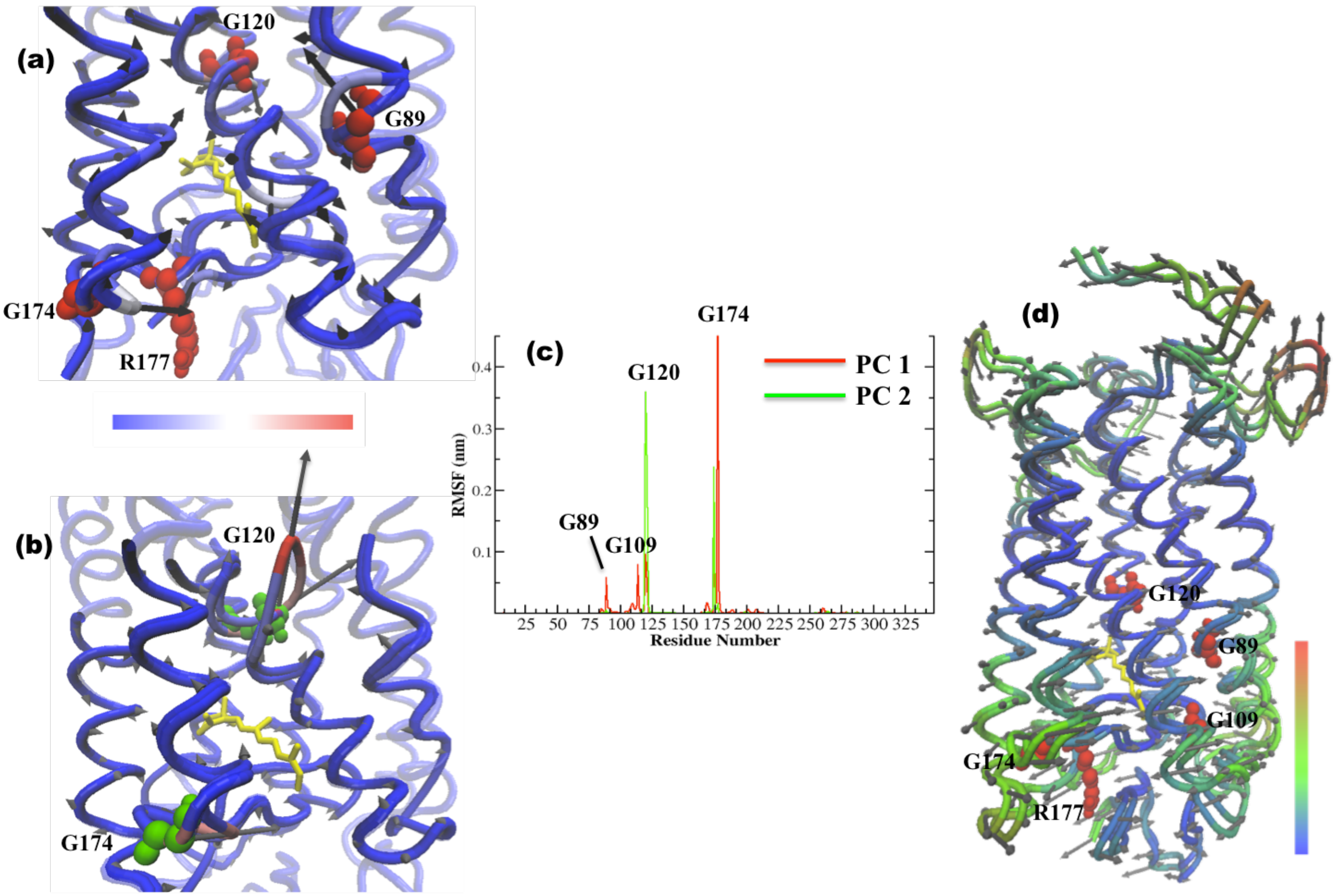

Figure S4: The most dominant modes of fluctuation derived from a PCA of the force trajectory of dark-state rhodopsin are mapped onto a C-α representation of the receptor. The arrows show the direction and amplitude of the extreme residue positions when projecting along the (a) first or (b) second eigenvector. The C-α representation of the receptor is color coded to reflect the amplitudes of the induced fluctuations. Regions in blue are less mobile and regions in red are more mobile. (c) Root mean square fluctuations (RMSF) of residues of PC1 and PC2 from the PCA-force distribution analyses. (d) The dominant PCA mode from the MD simulation of the dark-state of rhodopsin. The areas colored in red describe regions that are more mobile and regions in blue illustrate regions that are less mobile.

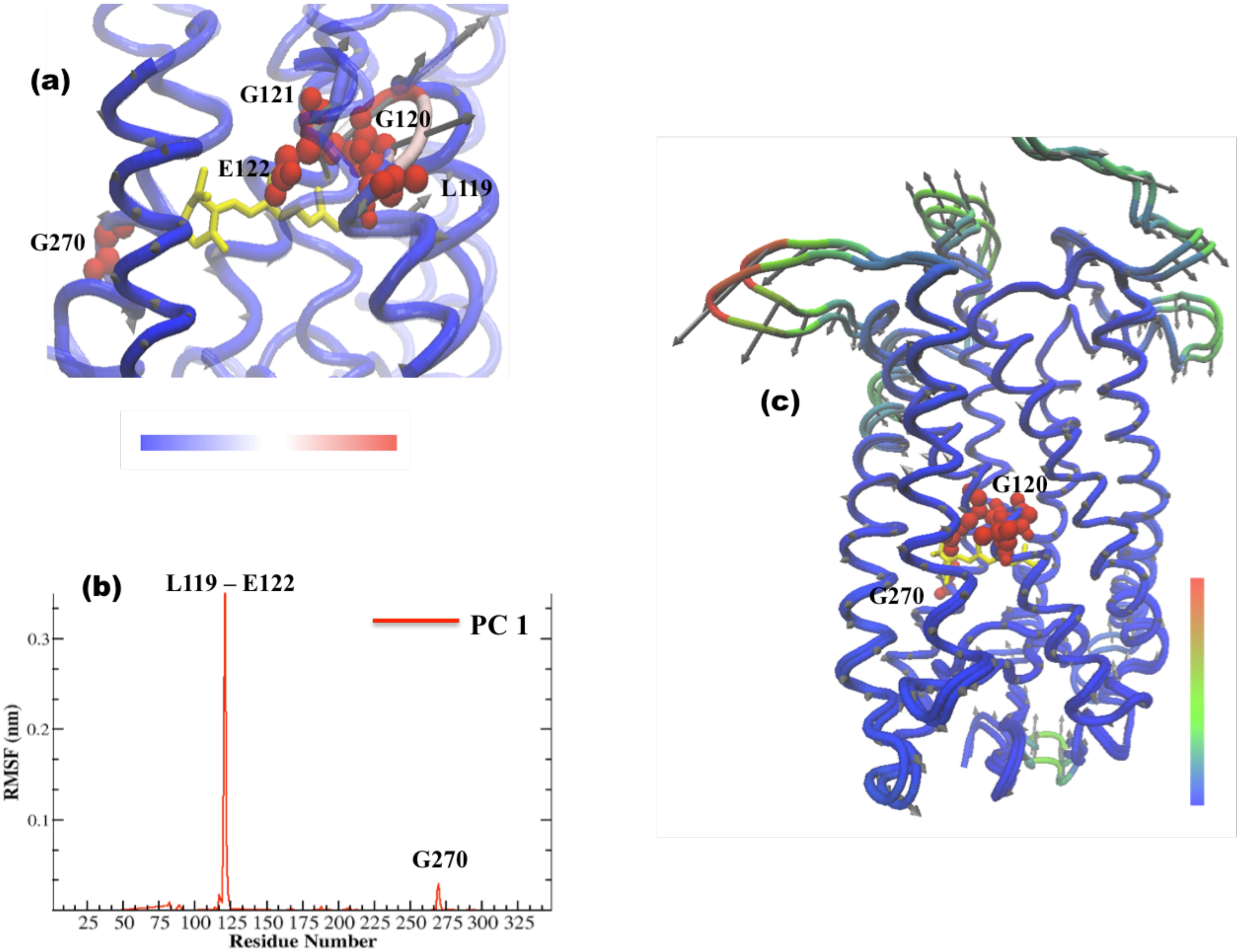

Figure S5: The most dominant mode of fluctuation derived from a PCA of the force trajectory of Meta II rhodopsin is mapped onto a C-α representation of the receptor. The arrows show the direction and amplitude of the extreme residue positions when projecting along the (a) first eigenvector. The C-α representation of the receptor is color coded to reflect the amplitudes of the induced fluctuations. Regions in blue are less mobile and regions in red are more mobile. (b) Root mean square fluctuations (RMSF) of residues from the PCA-force distribution analysis. (d) The dominant PCA mode from the MD simulation of Meta II rhodopsin. The areas colored in red describe regions that are more mobile and regions in blue illustrate regions that are less mobile.

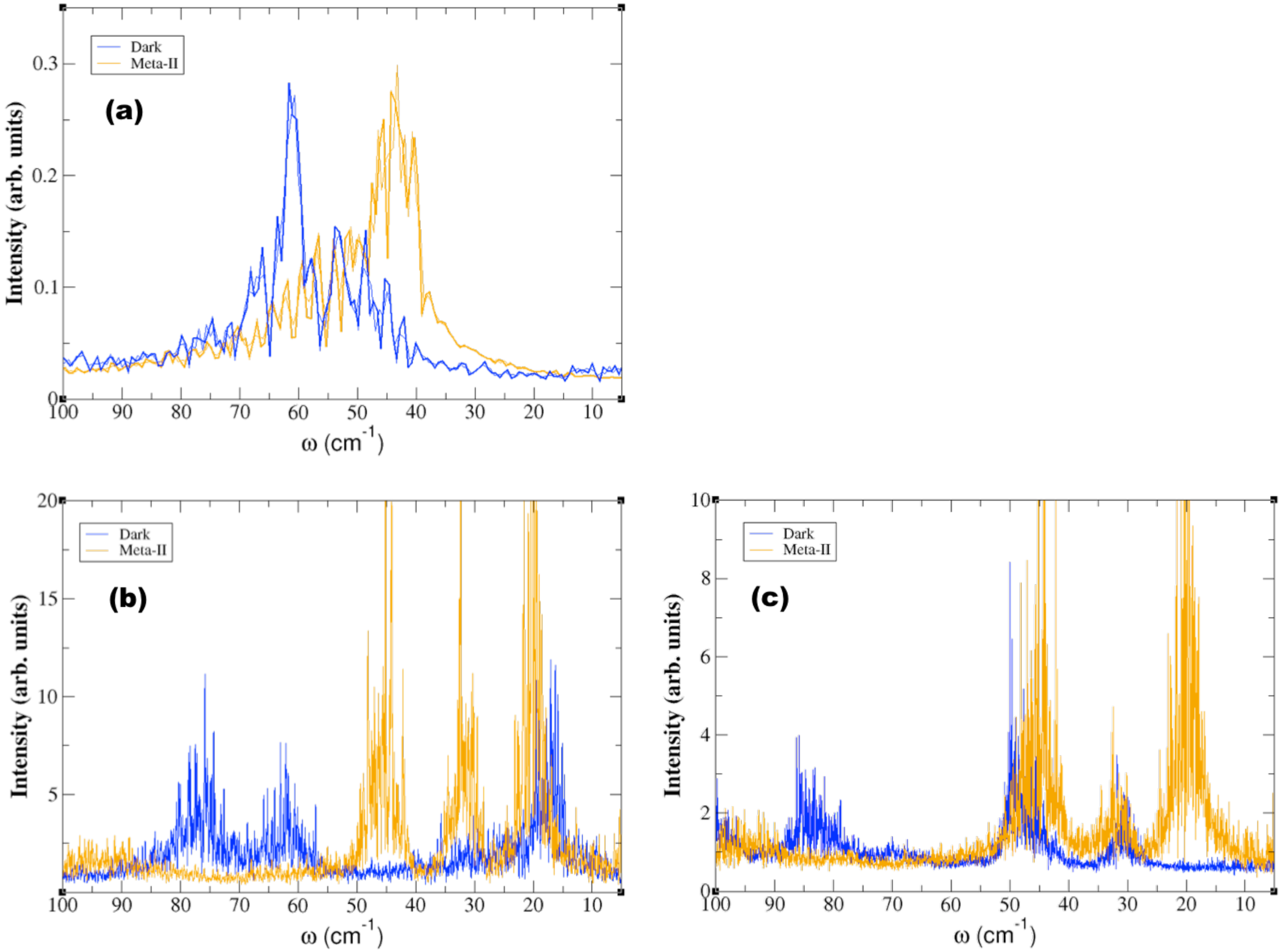

Figure S6: (a) Fourier transform of the velocity autocorrelation function of the backbone atoms of the dark-state (blue) and Meta II state (orange) of rhodopsin from the MD simulation. (b) Fourier transform of the force-induced time correlation function of backbone atoms of residues in contact with the retinal from the FDA. (c) Fourier transform of the force-induced time correlation function of side-chain atoms of residues in contact with the retinal from the FDA.

Figure S7: (a) Cartoon representation of rhodopsin in the inactive state from the MD simulation. The residues labeled show a conserved network of H-bonds consisting of structural water molecules and conserved polar residues. The D(E)RY, CWxP, Schiff base-counter ion and NPxxY network motifs are shown in red, blue, green and magenta licorice representation, respectively. The CWxP motif is believed to convert between two different "rotamer conformations" in the inactive and active states. (b) The spectrum of the fluctuations from the MD simulation of inactive rhodopsin showing the rotamer torsion of the CWxP motif(blue), torsional oscillation of the retinal (orange), vibrational fluctuation of Asn302 vibrational network that forms the NPxxY motif (magenta), and the vibrational spectrum of water molecules (red) that are part of the conserved network of H-bonding residues depicted in (a).

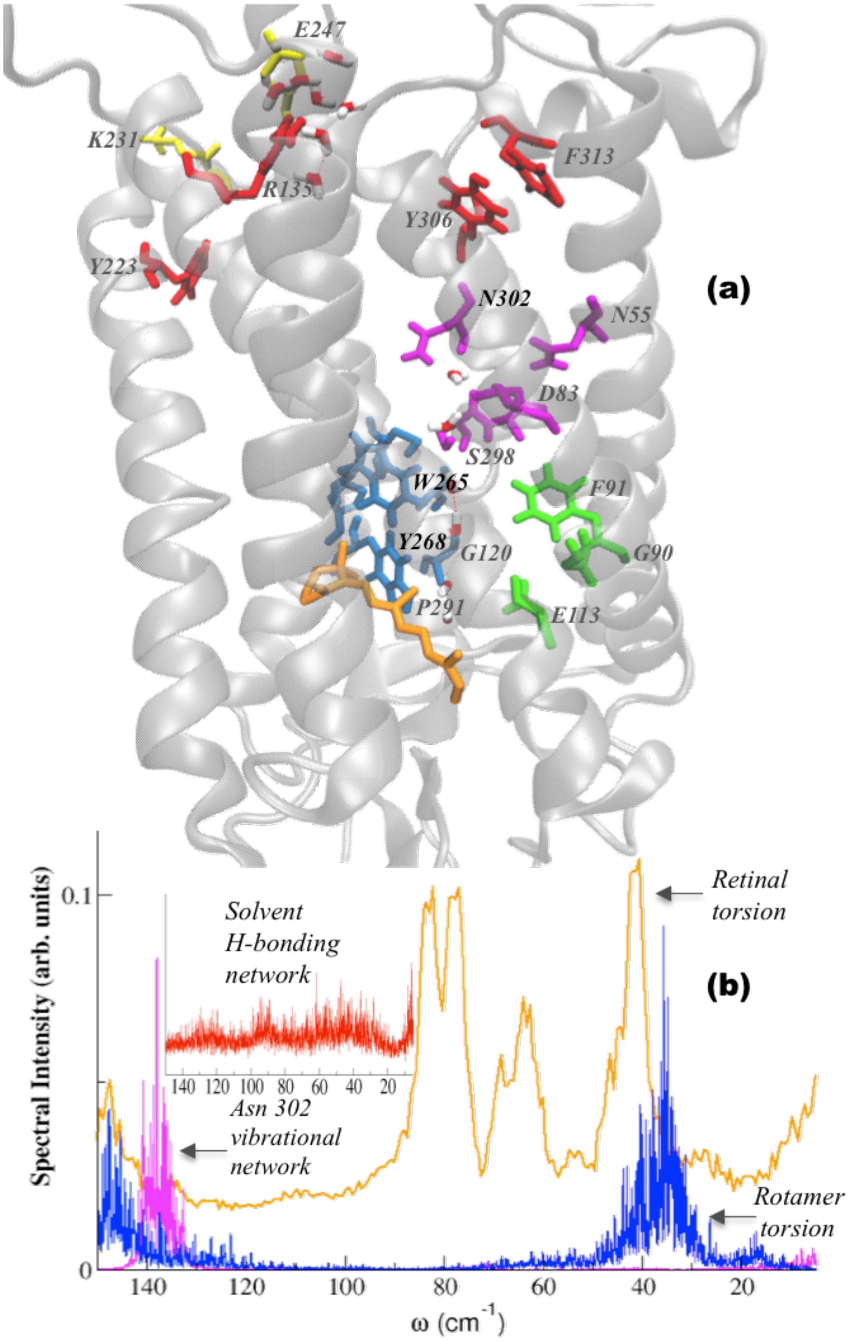

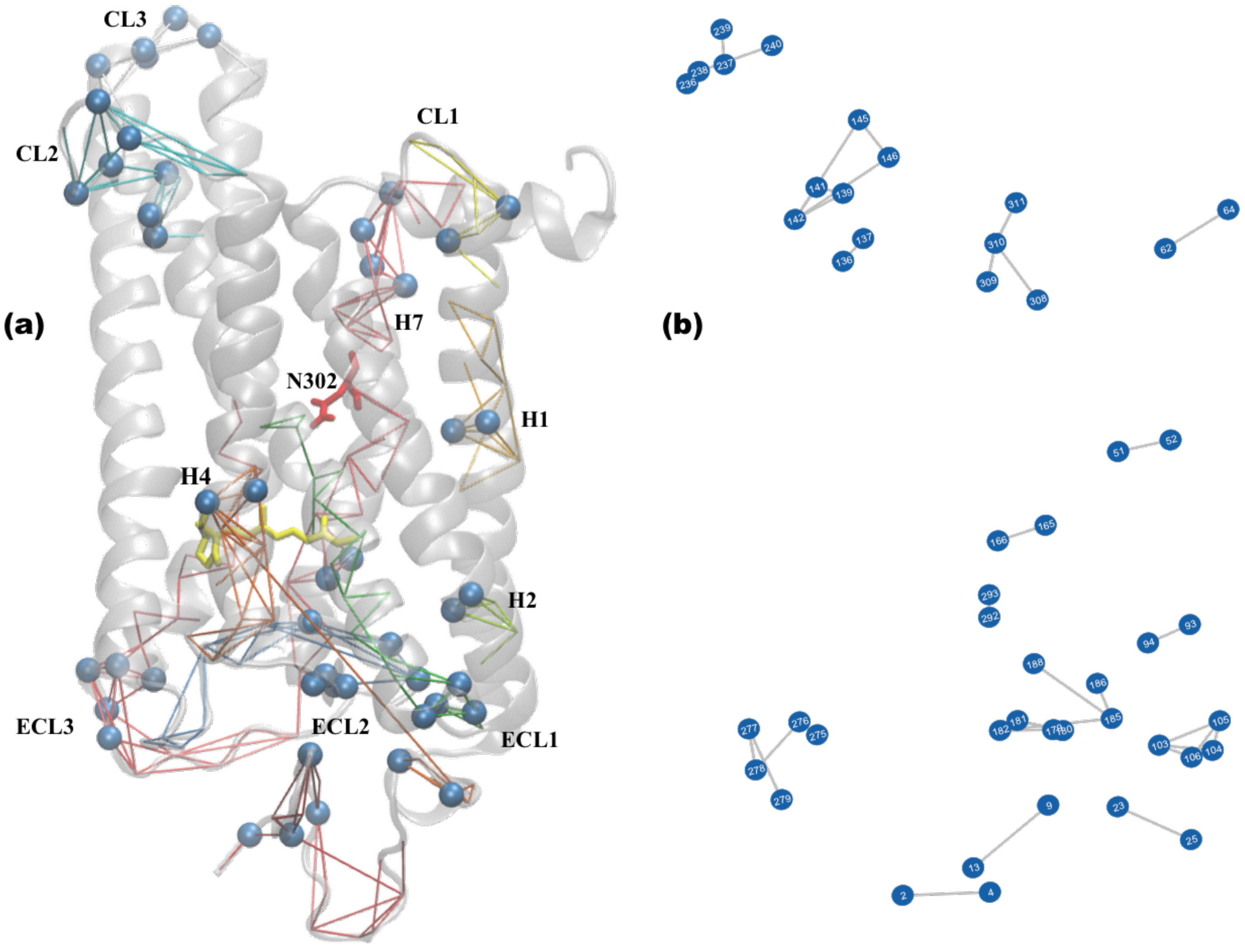

Figure S8: (a) Cartoon representation of Meta II rhodopsin showing the mapping of the LSFs from the MD simulation onto the protein 3-D structure. The figure also shows the overlap of the global structural fluctuations (GSFs) with the LSFs in Meta II. The different colors in the edges of the LSF network mapping represent regions of correlated fluctuations. The blue nodes represent GSF amino acid residues that are maximally overlapped with the LSFs. (b) 2-D network representation of the GSF nodes.

Supplementary Signal propagation and GSFs*:* Signal propagation and global fluctuations in Meta II (Figure S8)

A mapping of the LSFs (localized structural fluctuations) with the GSFs (global structural fluctuations) from the MD simulation allows us to investigate the relationship (overlap) between the local conformational fluctuations taking place in the receptor and the global, collective motions (as seen in Fig. S5c) that accompany activation. We find that the change in interactions surrounding the retinal β-ionine ring during activation moves it closer to the extracellular side where it has closer contact with residues in helix 4 (particularly Cys167 – Ala169). The new interactions create a long-distance collective torsion that links the helix 4 residues with residues in the N-terminus (Pro23 – Glu25) and provides the necessary space for the helix 6 outward rotation and the CL3 loop connecting helices 5 and 6. The changes in retinal interactions in the active-state of the receptor also modify contacts with residues that regulate interhelical packing within the retinal ligand-binding pocket. Particularly, the global elongation torsion of Meta II alters the tertiary contacts of residues involved with conserved water-mediated interactions in helices 1 and 2 such as Gly51, Phe52, Thr92, and Thr93. The modifications in the tertiary contacts[4] in the receptor hydrophobic core are a necessary precursor for signal propagation and are a consequence of the counterion switch from Glu113 to Glu181 in the active-state receptor. The global elongation torsion is also coupled with localized fluctuations that move the signal from the retinal ligand-binding pocket out toward the G-protein coupled region. Specifically, residues in EL1 -EL3 and the N- terminus are correlated with the dynamics of residues in helix 7 (Ala292 – Phe293, Met308 – Lys311) in the global torsion. The Asn302 H-bonding network of fluctuations lies between the two distinct segments of correlated fluctuations in helix 7 at the hinge of the torsion. The fluctuations of the Asn302 conserved H-bonding network connect the fluctuations in helix 7 near the retinal-binding pocket with the fluctuations in helix 7 that boarder the G-protein region in the IC domain via correlated torsional fluctuations through the hinge region of the receptor. Together, the coupled set of fluctuations in helix 7 move the excitation signal from the retinal ligand-binding region towards the intracellular side of the receptor and culminate as prominent outward fluctuations of loops in CL1, CL2, and the C-terminus.

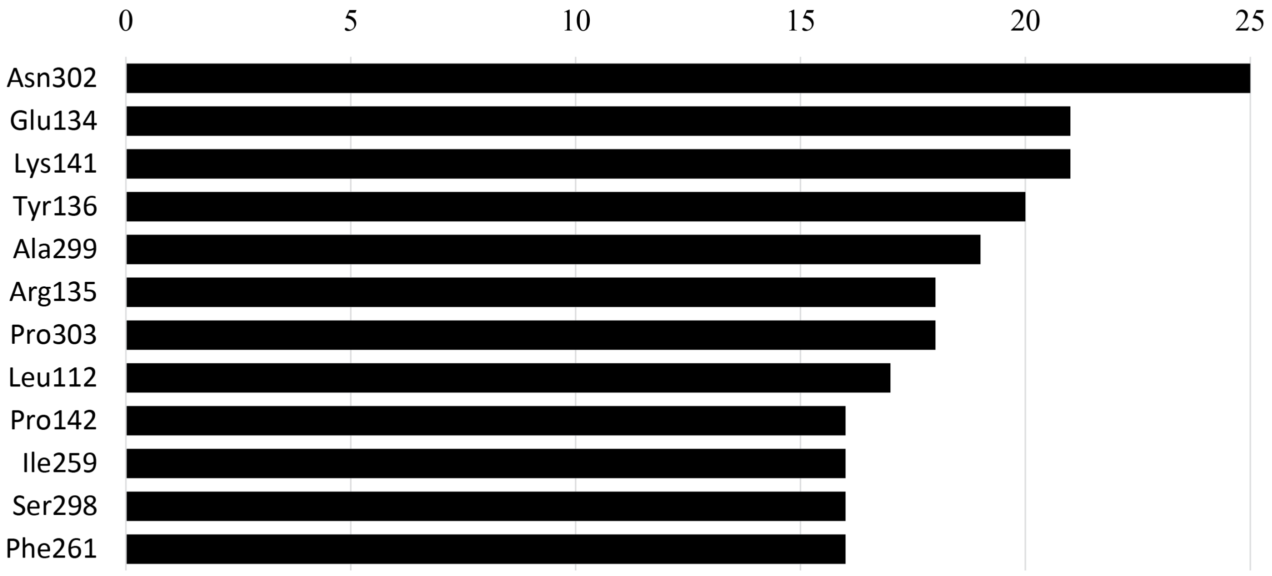

Figure S9: Top 12 nodes with the largest number of connections (links) from the MSA network in Figure 6. The graph lists the residues and the number of connections that each residue has in the MSA.

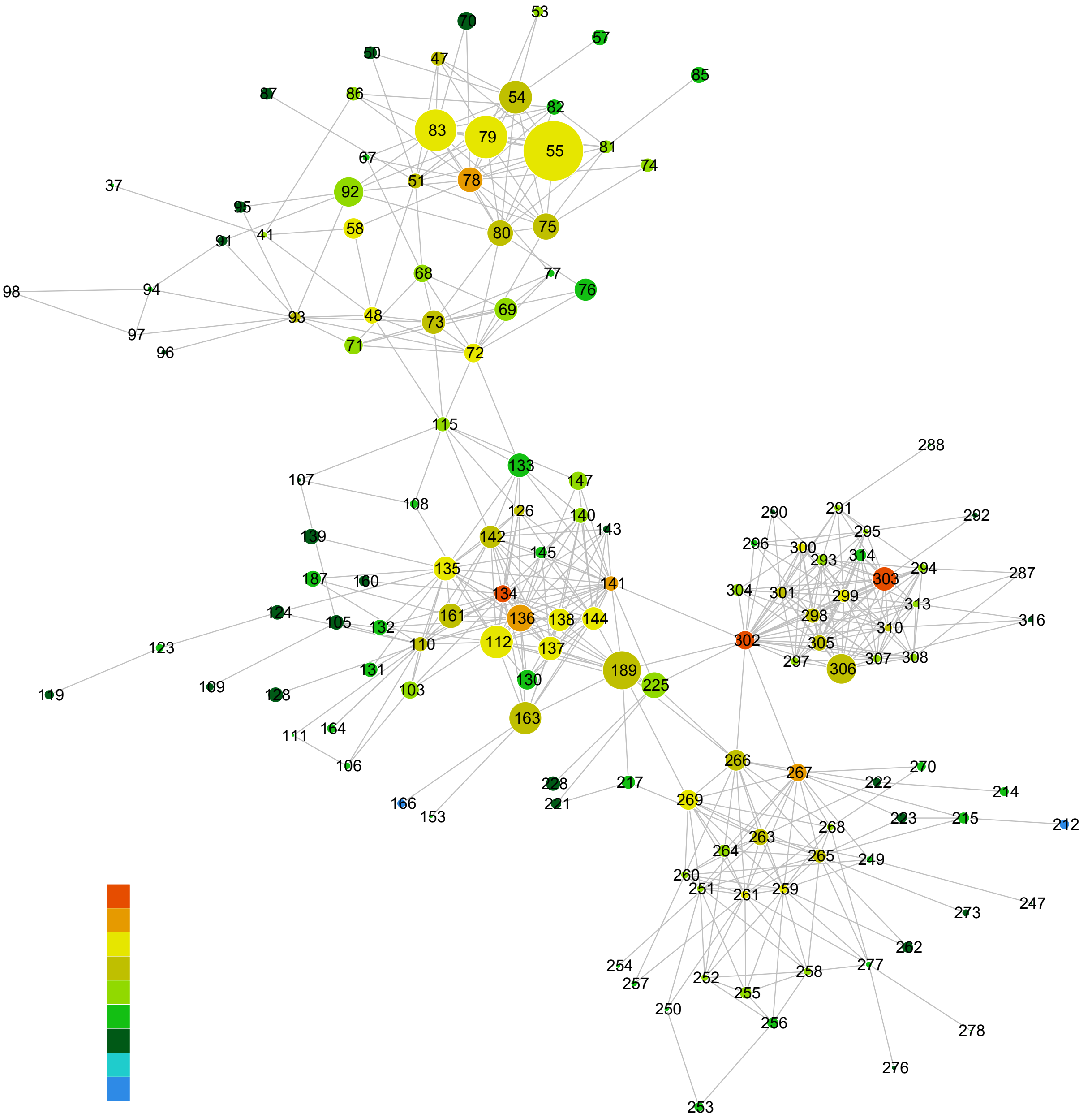

Figure S10: (a) MSA of the rhodopsin-like family of proteins. Nodes represent a specific amino acid and edges represent the lines between the nodes. The rainbow coloring of the nodes characterizes the degree to which a given amino acid takes part in the MI network (denoting coevolution propensity). Red nodes have a high MI value and blue nodes a low MI value. The size of the nodes in this case corresponds to the conservation of the amino acid from the MSA. The reference structure for the MSA is bovine rhodopsin (opsd_bovin) with pdb ID 1u19.

Materials and Methods

Force Distribution Analysis (FDA)

A modified version of Gromacs 4.5.3 was used to write out the pair-wise forces[1], Fij, between each residue pair i and j. Forces include contributions from the electrostatic and van der Waals interactions involving the retinal and rhodopsin that are calculated below a certain cut-off distance. These non-bonded pairwise forces of the residue pairs in close proximity comprise a force-propagation network involving short-range to medium-range connections that are averaged over the simulation time. The averaged forces were saved every 10 ps and convergence was reached when an equilibrium value for the forces was attained. The stored forces were written out as force trajectories and the average of those forces were used later for further analysis in R (https://www.r-project.org/) as well as visualization in VMD (http://www.ks.uiuc.edu/Research/vmd/). Covariance matrices and principal component analyses (PCA) were used on the averaged residue forces to identify correlated changes in the pair-wise forces in both the dark- and Meta II state of rhodopsin. Eigenvalues and eigenvectors were calculated by diagonalizing the covariance matrix, and eigenvectors were sorted in descending order of their eigenvalue. In each case, > 80% of the variance was described in either the top two or top PCA mode in the dark- and Meta II state, respectively. The modes of fluctuation were visualized by calculating a trajectory along either the first two eigenvectors in the dark-state or the first eigenvector in the Meta II state.

References

1. Stacklies, W., Seifert, C. & Graeter, F. Implementation of force distribution analysis for molecular dynamics simulations. *BMC Bioinformatics* **12,** 101 (2011).
2. Kubo, R., Toda, M. & Hashitsume, N. *Statistical Physics II: Nonequilibrium Statistical Mechanics*. (Springer Science & Business Media, 2012).
3. Stacklies, W., Vega, M. C., Wilmanns, M. & Gräter, F. Mechanical Network in Titin Immunoglobulin from Force Distribution Analysis. *PLoS Comput Biol* **5,** e1000306 (2009).
4. Yamazaki, Y. *et al.* Intramolecular interactions that induce helical rearrangement upon rhodopsin activation: light-induced structural changes in metarhodopsin IIa probed by cysteine S-H stretching vibrations. *J. Biol. Chem.* **289,** 13792–13800 (2014).
